# Dual Effect of *EZH2* Gene Editing with CRISPR/Cas9 in Lung Cancer

**DOI:** 10.3390/biology15030251

**Published:** 2026-01-29

**Authors:** Joice M. Menezes, Diego C. de Mello, Kelly C. Saito, Edna T. Kimura, Cesar S. Fuziwara

**Affiliations:** 1 Department of Cell and Developmental Biology, Institute of Biomedical Sciences, University of São Paulo, São Paulo 05508-000, SP, Brazil; jmmenezesbio@icb.usp.br (J.M.M.); mellodc@usp.br (D.C.d.M.); saito@icb.usp.br (K.C.S.); etkimura@usp.br (E.T.K.); 2 Department of Biochemistry, Escola Paulista de Medicina, Universidade Federal de São Paulo, São Paulo 04044-020, SP, Brazil

**Keywords:** *EZH2*, CRISPR/Cas9, lung cancer, gene editing, Polycomb group genes, EPZ6438, mouse model, tumor progression, EMT

## Abstract

Lung adenocarcinoma is the most common form of lung cancer with a 5-year survival rate of 15%, largely due to asymptomatic metastasis and late diagnosis. Overexpression of Polycomb group (PcG) proteins, particularly EZH2, the catalytic component of Polycomb Repressive Complex 2 (PRC2), has been associated with the pathogenesis of lung cancer, frequently showing correlation with cancer progression and poor prognosis. In this study, EZH2 levels were modulated by CRISPR/Cas9 gene editing and PRC2 activity was inhibited with EZH2 inhibitor EPZ6438 or EED inhibitor MAK683. *EZH2* gene editing reduced cell proliferation, migration, invasion, and colony formation and reduced NFκ-B signaling activation, indicating an antitumoral effect in vitro. Moreover, EZH2 inhibition also increased the expression of differentiation-related genes, such as *GATA5*, *FOXA2*, and lung surfactants, indicating a pro-differentiation effect. However, EZH2-edited cells injected into an immunocompromised mouse model generated larger tumors compared to unedited cells. This was accompanied by increased expression of other PcG genes, including *EZH1*, *CBX2*, *RING1*, *EED*, and *SUZ12*, suggesting a compensatory interaction between PRC2 and PRC1 complexes. These findings provide significant clinical relevance, both in elucidating the mechanisms of novel molecular targets and in guiding treatment strategies for lung cancer when using epigenetic inhibitors.

## Simple Summary

Searching for new molecular targets for lung cancer is essential to understand aggressiveness and early metastatic spread and provide new treatment alternatives. This study indicates a dual and cell context-dependent role for EZH2 activation and points out the challenges of targeting this epigenetic regulator in lung cancer. Our findings demonstrate that EZH2 inhibition induces tumor suppressor effects in vitro, promoting cell differentiation, but can trigger compensatory epigenetic mechanisms that promote tumor progression in vivo. This study highlights the plasticity of the networks regulated by Polycomb group (PcG) genes, raising important considerations for the use of epigenetic therapies in lung cancer, including EZH2 inhibitors or even *EZH2* gene editing, either alone or in combination with the inhibition of other PcG genes.

## 1. Introduction

Lung cancer is among the most frequently diagnosed cancers and is the leading cause of cancer death [1]. In the USA, in 2026, it is estimated that 229,410 new cases of lung cancer will be diagnosed and, of these, 124,990 will result in death (~54%) [2].

Non-small cell lung cancer (NSCLC) accounts for over 85% of all lung cancer cases, and lung adenocarcinoma (LUAD) is the most frequently diagnosed subtype, which represents approximately 40% of NSCLC cases [3]. Several treatment strategies have been developed for NSCLC patients; however, the long-term prognosis is still poor, with an overall 5-year survival rate of less than 15%, mainly due to late-stage detection and limited availability of treatments for advanced stages [4]. Additionally, in lung cancer, approximately 53% of patients are diagnosed with distant metastases, primarily affecting the bones, liver, and brain, resulting in an 8% survival rate for patients with metastatic disease (SEER National Cancer Institute-EUA) [5].

The main oncogenic pathway in lung cancer is MAPK, with mutations in *KRAS* (~33%) and *EGFR* (~14%), and high prevalence of inactivating mutations in *TP53* (~46%) in adenocarcinomas [6]. Moreover, large-scale genome sequencing of patients with adenocarcinoma revealed additional genetic alterations in *CDKN2A* (~12.6%), *ALK* (~10.2%), *RET* (~6%), and *BRAF* (~2%) [7,8,9]. However, recent advances and efforts in early detection and clinical diagnosis of lung cancer have failed in significantly reducing the high mortality rates linked to this disease. In this context, investigating new molecular alterations in lung oncogenesis could improve our understanding of the biology of this cancer.

Polycomb group (PcG) genes are a class of epigenetic regulators that play critical roles in the maintenance of cellular identity [10]. PcG proteins function in several families of multiprotein complexes, such as the EZH2/PRC2 complex (Polycomb Repressive Complex 2/Enhancer of Zest Homolog 2). In the canonical pathway, PRC2 catalyzes the di- and tri-methylation of lysine 27 on histone H3 (H3K27me2/3) via enzymatic activity of EZH2 [11,12]. Data from TCGA (The Cancer Genome Atlas) show that increased expression of *EZH2* is associated with worse prognosis in lung cancer [13], and negatively correlates with patient survival. In addition, elevated *EZH2* expression is also observed across different types of cancer, such as prostate, breast, stomach, bladder, and brain cancer, and is related to increased cell proliferation, invasion, and motility [14].

Although EZH2 was initially identified as the sole histone methyltransferase of PRC2, recent studies have shown that EZH1 is part of a noncanonical PRC2 complex that catalyzes the methylation of H3K27 and prevents the derepression of PRC2 target genes [15,16]. The catalytic activity of both EZH1 and EZH2 is contingent on their formation of a complex with SUZ12 and EED [17]. Thus, studying the role of PcG genes could bring important advances in understanding the biology of lung cancer.

In this study, we investigated the role of EZH2 in lung adenocarcinoma. For that, EZH2/PRC2 function was modulated by gene with CRISPR/Cas9 and pharmacologically inhibited with the EZH2 inhibitor EPZ6438 or the EED inhibitor MAK683. The main results showed an antitumoral effect of *EZH2* gene editing in lung adenocarcinoma cells in vitro. However, *EZH2*-edited cells injected into immunocompromised mice exhibited a substantial increase in tumor growth, linked to an increase in other PcG genes such as *EZH1*, *EED*, *SUZ12*, *CBX2,* and *RING1*, suggesting a cooperative and compensatory effect between the complexes upon EZH2 inhibition. Blocking EED pharmacologically exerted reinforced antitumoral effects in *EZH2*-edited cells. These findings highlight that the impact of EZH2 inhibition depends on the cellular context, raising important considerations for the use of epigenetic therapies in lung cancer, including pharmacological inhibitors or gene editing of *EZH2*, either alone or in combination with the inhibition of other PcG genes.

## 2. Materials and Methods

### 2.1. Cell Culture and Treatments

#### 2.1.1. Cell Culture

The main cell line used was A549 derived from human lung adenocarcinoma. We used the lung cancer cell lines Calu3 and H2122 for gene expression and Western blot assays. The corresponding cell culture medium, histology, genetic drivers, and identifiers are shown in Supplementary Table S1. All cells were maintained in an incubator at 37 °C and 5% CO_2_. These cell lines (A549, Calu3, and H2122) were donated by Dr. Lin He from University of California Berkeley in 2019. The cell line origin as lung cancer was authenticated using short tandem repeat (STR) analysis following the ANSI Standard ASN-0002-2012, and the results were compared with the DSMZ and Cellosaurus databases. Briefly, 16 STR loci plus the gender-determining locus Amelogenin were amplified using the commercially available PowerPlex^®^ Fusion 6C Kit (Promega, Madison, WI, USA). Samples were processed on an ABI 3130 Genetic Analyzer (Thermo Fisher Scientific, Waltham, MA, USA), and data were analyzed with GeneMarker^®^ hid software v 2.8.2 (SoftGenetics, State College, PA, USA). In addition, all experiments were performed using mycoplasma-free cells.

#### 2.1.2. EZH2 Pharmacological Inhibition

The A549 and H2122 cell lines were treated with EPZ6438 (Tazemetostat-Cayman Chemical, Ann Arbor, MI, USA), an allosteric inhibitor of EZH2 methyltransferase activity, at 5 µM concentration for six days, with drug supplementation every other day. As a control, A549 and H2122 cells were treated with DMSO (vehicle) at 0.001% in culture medium.

#### 2.1.3. EED Pharmacological Inhibition

The A549 cell line was treated with MAK-683 (Selleck Chemicals, Houston, TX, USA), an inhibitor that binds to EED and disrupts the PRC2 complex by reducing H3K27me3 levels. The drug was used at 2 µM concentration for six days, with drug supplementation every other day. As a control, A549 cells were treated with DMSO (vehicle) at 0.001% in culture medium.

### 2.2. EZH2 Gene Editing with CRISPR/Cas9

To achieve permanent suppression of EZH2 expression in A549 cells, we employed the CRISPR/Cas9 system using sgRNAs previously cloned into pSpCas9(BB)-2A-Puro (PX459) puromycin resistance plasmids named as PX459-*EZH2*-sg7, PX459-*EZH2*-sg18, and PX459-*EZH2*-sg25 (Supplementary Table S2). Briefly, 1 µg of plasmids were transfected in A549 cells using Lipofectamine^TM^ 2000 reagent (Invitrogen, Thermo Fisher Scientific, Waltham, MA, USA), and stable populations were selected with 1 µg/mL of puromycin for 7 days. An empty vector of PX459 was used to create A549-CTR cells. We generated single-cell CRISPR-knockout clones by limiting dilution plating as previously described [18].

After extraction of genomic DNA using a DNeasy Blood & Tissue Kit (Qiagen, Hilden, Germany), we PCR-amplified and sequenced, by the Sanger method, the genomic region targeted by sg25 to validate the gene editing specificity and the segment of potential off-targets in *WDR7* and *DCLK3* genes using specific primers (Supplementary Table S2). To determine gene editing efficiency, we analyzed the sequencing files (.ab format) from A549-CTR and A549-edited cells in the SeqScreener Gene Edit Confirmation App (SGC—https://apps.thermofisher.com/apps/gea-web, accessed on 6 January 2026).

### 2.3. Cell Function Assays

#### 2.3.1. Cell Counting

Cell growth was assessed by seeding 5 × 10^4^ cells per well in 6-well plates and incubating for 24, 48, and 72 h. At each time point, cells were washed with PBS, detached using trypsin, fixed in 3.7% formaldehyde, and collected into fresh tubes. Cell counts were performed in triplicate using a Guava EasyCyte Mini cytometer (Millipore, Burlington, MA, USA) to determine the average number of cells, as previously described [18].

#### 2.3.2. Mitochondrial Activity Assay (MTT)

Determination of mitochondrial activity was performed as previously described [19]. Briefly, cells were plated at 2 × 10^4^ cells per well in 96-well plates and allowed to adhere for 24 h. Next, 3-(4,5-dimethylthiazol-2-yl)-2,5-diphenyltetrazolium bromide (MTT; Molecular Probes, Eugene, OR, USA) was added to the cell culture medium to a final concentration of 250 μg/mL and incubated at 37 °C in a 5% CO_2_ incubator for 4 h. The medium was then removed, and 100 μL of isopropanol/0.01 M HCl was added to dissolve the formazan crystals. Absorbance was measured at 595 nm using a SpectraMax M plate reader (Molecular Devices, San Jose, CA, USA).

#### 2.3.3. Cell Invasion and Migration

We used Transwell chambers (Corning Inc., New York, NY, USA) either precoated with 50 µL of Geltrex™ Matrix (Gibco, Thermo Fisher Scientific, Waltham, MA, USA) for an invasion assay or left uncoated for a migration assay, as previously described [18]. In the invasion assay, 5 × 10^4^ A549-CTR and A549-sg25 cells were seeded in the upper chamber in triplicate in medium containing 0.5% FBS, while the lower chamber contained medium with 10% FBS as a chemoattractant. After 48 h, non-invading cells on the upper surface of the membrane were removed with a cotton swab, and the invading cells were fixed in 3.7% formaldehyde in PBS and stained with 1% crystal violet in 2% ethanol. For the migration assay, 2.5 × 10^4^ cells were seeded under the same conditions and were collected after 24 h. Membranes were processed as described for the invasion assay. Then, the membrane was removed from the insert and mounted into a glass slide and images were acquired using a Nikon Eclipse E600 microscope (Shinagawa, Tokyo, Japan) equipped with a CF160 epifluorescence camera, and the number of stained cells was quantified at 100× magnification using ImageJ software v.1.53o.

#### 2.3.4. Colony Formation

To assess the clonogenic ability, we proceeded as previously described [18]. Briefly, 300 cells were seeded in triplicate in 6-well plates and cultured for 8 days. Colonies were then fixed with 3.7% formaldehyde and stained with 1% crystal violet in 2% ethanol. Representative images of replicates were captured, and the total colony area was quantified using ImageJ software.

#### 2.3.5. Suspension Culture

Six-well plates were coated with 1 mL of 1.2% of poly (2-hydroxyethylmethacrylate) polymer (poly-HEMA) (Sigma, St. Louis, MO, USA) solution diluted in 95% ethanol and left to dry at room temperature under UV light with no lid for 16 h in a laminar flow hood. Plates were washed twice with PBS; then, 5 × 10^4^ A549-CTR and A549-sg25 cells were plated in triplicate into the poly-HEMA-coated wells in complete medium and cultured for 12 days. Cells were fixed with 3.7% formaldehyde, and representative images were taken under an inverted microscope EVOS XL (Thermo) from two independent experiments performed in triplicate.

### 2.4. Gene Expression Analysis

Total RNA was isolated and extracted according to the TRIzol protocol (Invitrogen) and proceeded as previously described [18]. To assess the expression of protein-coding genes, the cDNA was obtained using 1–3 µg of total RNA, oligo-dT primer, random primer, and MMLV reverse transcriptase (Invitrogen), following DNase treatment (Sigma). The expression of PRC2 components, lung differentiation genes and transcription factors, and EMT markers was analyzed by quantitative PCR (qPCR) using SYBR Green Master Mix, cDNA, and specific primers (Supplementary Table S2) on a ViiA7^®^ Sequence Detection System (Applied Biosystems, Thermo Fisher Scientific, Waltham, MA, USA). Gene expression was normalized by comparison with *ACTB* levels and calculated using the QGene program v.1 [20] using the Ct values.

To evaluate the expression of microRNAs, 10 ng of total RNA was reverse transcribed with the TaqMan^®^ Reverse Transcription Kit (Applied Biosystems, Thermo Fisher Scientific, Waltham, MA, USA) using stem-loop primers *hsa-miR-200a-3p* (assay 502), *hsa-miR-200c* (assay 2300), and *RNU6B* (assay 1093) for normalization (Applied Biosystems, Thermo Fisher Scientific, Waltham, MA, USA). qPCR was performed using the TaqMan Universal PCR Master Mix, No AmpErase^®^ UNG (Life Technologies, Thermo Fisher Scientific). Reactions were run on a ViiA7^®^ System, and miRNA expression values were normalized to *RNU6B* and calculated using QGene.

### 2.5. RNA-Seq Data Analysis

#### 2.5.1. Library Construction and Sequencing Analysis

Cells were lysed using TRIzol (Invitrogen) and total RNA was purified using the RNeasy Mini Kit (Qiagen) following the manufacturer’s instructions. RNA-seq was performed at the Central Laboratory of High-Performance Technologies in Life Sciences of the State University of Campinas (LaCTAD UNICAMP, Campinas, SP, Brazil). Truseq Stranded Total RNA–Ribo-zero Plus rRNA Depletion (Illumina, San Diego, CA, USA) was used for ribosomal RNA (rRNA) depletion, and library preparation was performed in triplicate for each cell line. Final sequencing was performed on an Illumina Nextseq 2000 with paired-end 2 × 100 bp. RNA-seq libraries were sequenced, generating 29 to 39 million reads for A549-CTR and 26 to 30 million reads for A549-sg25. Quality analysis, filtering, and trimming of library reads were performed using FASTQC and Trimmomatic. Reads were mapped to the *Homo sapiens* reference genome (Ensembl GRCh38 version 109) using bowtie2 (v2.4.4). Gene expression levels were then calculated using RSEM (v1.3.3), yielding values in counts, TPM (Transcripts Per Million) and FPKM (Fragments Per Kilobase of transcript per Million mapped reads). Reads were imported into R statistical software (v4.0) and differentially expressed gene analysis was performed using DESeq2 (v1.30.1). The analysis was based on normalized data and identified differentially expressed genes (DEGs) using an adjusted p-value (padj) of 0.05. For bioinformatic analysis, we selected a list of DEGs based on the Log_2_ (fold-change) value: ±1.

#### 2.5.2. Gene Ontology

The lists of DEGs were entered into the enrichment analysis tool Enrichr (https://maayanlab.cloud/Enrichr/, accessed on 13 January 2024) and we analyzed processes relevant to lung cancer, such as KEGG (Kyoto Encyclopedia of Genes and Genomes 2021 Human) pathways and gene ontology for Biological Process 2023 (BP), Cellular Component 2023 (CC), and Molecular Function 2023 (MF). Significantly enriched processes related to lung cancer (*p*-value < 0.05) were manually selected.

#### 2.5.3. Protein–Protein Interaction Networks

The STRING database (https://string-db.org/, accessed on 13 January 2024) was used to characterize the functional roles of DEGs and to construct protein–protein interaction networks among DEGs. We used a high confidence (>0.7) score in physical protein interactions as the cutoff criteria. We used Cytoscape (version 3.9.1) to visualize data and perform additional GO annotations and KEGG annotations of significant processes (*p*-value < 0.05). To identify important hub genes networks, we used the Molecular Complex Detection [21] plugin with the following criteria: Max. Depth = 100, K-Core = 2, Mode Score Cutoff = 0.2, and Degree Cutoff = 2. For representation, clusters and hubs relevant to lung cancer biology were selected.

### 2.6. Protein Extraction and Western Blot

Total protein extracts and Western blots were performed as previously described [19]. Protein was obtained from whole-cell lysates using RIPA buffer (20 mM Tris, pH 7.5, 150 mM NaCl, 1% Nonidet P-40, 0.5% sodium deoxycholate, 1 mM EDTA, and 0.1% SDS) containing 10% protease inhibitor cocktail, and concentration was determined via the Bradford assay (Bio-Rad Laboratories, Hercules, CA, USA).

For Western blot, 30 µg of total protein per sample was resolved on 10% SDS–PAGE gels and transferred to Hybond-ECL nitrocellulose membranes (Amersham Biosciences, Little Chalfont, UK). Membranes were blocked with 5% skim milk in Tris-buffered saline containing 0.1% Tween-20 (TBS-T) to prevent nonspecific binding. Primary antibodies and incubation specifics are shown in Supplementary Table S3. Bound antibodies were detected using HRP-conjugated secondary antibodies, and signal development was achieved with luminol and p-coumaric acid reagents (Sigma) in the presence of hydrogen peroxide. Chemiluminescence was visualized using the ImageQuant LAS 4000 imaging system (GE Healthcare, Little Chalfont, UK), and band intensities were quantified with ImageJ software.

### 2.7. Luciferase Reporter Gene Assay

The transcriptional activities of the NF-κB, Wnt/β-catenin, TGFβ, and Notch pathways were evaluated using luciferase reporter constructs. The NF-κB pathway was analyzed using the pGL4.32 [luc2P NF-κB-RE Hygro] reporter (Promega, Madison, WI, USA), which contains five tandem NF-κB response elements upstream of the luciferase gene. Wnt/β-catenin signaling was assessed with the M50 Super 8× TOPFlash (pM50) vector, harboring seven TCF/LEF-binding motifs (AGATCAAAGGgggta). TGFβ activity was measured with the SBE4-Luc plasmid (a gift from Bert Vogelstein, Addgene plasmid #16495), which contain four Smad-binding elements (GTCTAGAC) cloned into the pBV-Luc backbone. The 4xwtCBF1Luc construct, containing four CBF1-binding sequences, was used to measure Notch pathway activation [22].

The experiment was performed as previously described [18]. Fifty thousand cells were plated in 24-well plates. After 24 h, cells were co-transfected with 300 ng of reporter plasmid plus 30 ng of pRL (*Renilla* luciferase-normalization control) (1:10 ratio) using Lipofectamine 2000^TM^ (Invitrogen) in the proportion of 1:2 (ug of DNA:uL of lipofectamine2000). After 4 h of transfection, the medium was changed to a complete medium. Twenty-four hours after transfection, the cell lysate was collected using 1x Passive Lysis Buffer (Promega, Madison, WI, USA, cat. E1500) and luminescence was measured using a Dual Luciferase^®^ Reporter Assay kit (Promega) in the luminometer GloMax (Promega).

### 2.8. In Vivo Functional Assay

#### 2.8.1. In Vivo Xenotransplant in Nude Mice

This study complied with the guidelines of the Institutional Animal Care and Use Committee (IACUC) of the Institute of Biomedical Sciences, University of Sao Paulo (ICB/USP), protocol number CEUA No. 2023150720.

For tumor xenografts, 2 × 10^6^ A549-CTR and A549-sg25 cells were suspended in cold PBS and mixed at a 1:1 ratio with Geltrex™ Matrix (Gibco) to a final volume of 100 µL. Each mixture was injected subcutaneously into opposite flanks of nude mice (*n* = 8), with A549-CTR on the left and A549-sg25 on the right side. Tumor progression was monitored for 35 days, and tumor volume (V) was determined using caliper measurements of length (L) and width (W) according to the formula V = (L × W^2^)/2.

At the endpoint, animals were euthanized by intraperitoneal injection of a ketamine/xylazine overdose (300 mg/kg and 30 mg/kg, respectively). Tumors were then excised, weighed, and fixed in 3.7% buffered formaldehyde, followed by sequential dehydration in ethanol (70–100%) and xylene perfusion prior to paraffin embedding in the LEICA TP 1020 Automatic Tissue Processor (Leica Biosystems, Nußloch, Germany) for subsequent histological and immunohistochemical (IHC) analysis.

#### 2.8.2. Histological and IHC Analysis

Paraffin-embedded tumors were sectioned into 3 μm slices. Sections were stained with hematoxylin and eosin (H&E) for morphological assessment. For IHC, we followed a previously established protocol [19] using 3 μm sections that were deparaffinized and rehydrated in PBS, and endogenous peroxidase activity was blocked by the incubation with 3% hydrogen peroxide. Slides were then incubated overnight at 4 °C with a rabbit anti-β-catenin antibody (Santa Cruz, Dallas, TX, USA, sc-7199; 1:100 dilution in TBS/BSA).

After washing, sections were incubated for 2 h at room temperature with a biotinylated anti-rabbit secondary antibody (Sigma; 1:250 in TBS/BSA), followed by another 2 h incubation with ExtrAvidin^®^–peroxidase (Sigma; 1:250). PBS washes were performed between all incubation steps. Immunostaining was visualized using DAB/BSA with H_2_O_2_ as the chromogen. Positive reactions appeared as brown cytoplasmic or nuclear precipitates, and staining intensity was scored as negative (–), weak (+), medium (++), and strong (+++).

### 2.9. Analysis of the Cancer Genome Atlas (TCGA) Data

EZH2 expression in lung cancer tissues retrieved from TCGA database was analyzed using GEPIA (http://gepia.cancer-pku.cn/index.html, accessed on 6 May 2024) from 59 paired non-tumor samples and 483 lung adenocarcinoma samples, and 50 paired non-tumor samples and 486 lung squamous cell carcinoma samples.

The overall survival rate of patients with lung adenocarcinoma (LUAD) and lung squamous cell carcinoma (LUSC), was analyzed from data retrieved from the Kaplan–Meier Plotter database (https://kmplot.com/analysis/, accessed on 6 May 2024) [23]. The analysis was performed in 1161 LUAD patients and 780 LUSC patients that were available in the database.

### 2.10. Statistical Analysis

The results were presented as the mean ± standard deviation and were submitted to an unpaired two-tailed Student’s *t*-test. Differences were considered significant at *p* ≤ 0.05.

## 3. Results

### 3.1. EZH2 Is Overexpressed in Lung Cancer and Associated with Poor Survival

**Figure 1:**
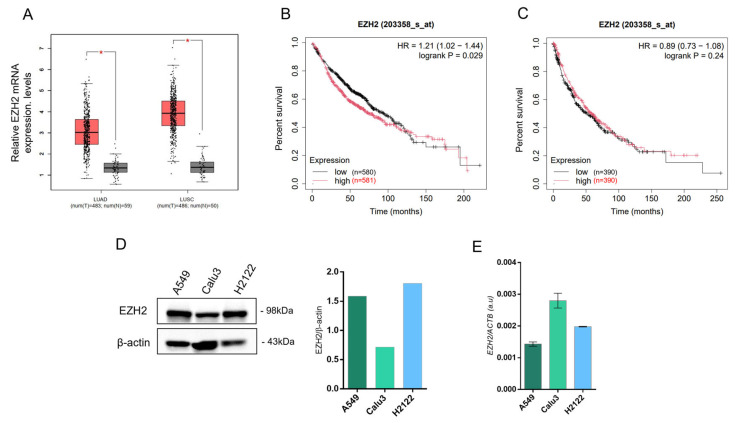
EZH2 is overexpressed in lung cancer and associated with poor survival. (**A**) The expression of *EZH2* in lung cancer tissues retrieved from TCGA database was analyzed using GEPIA (http://gepia.cancer-pku.cn/index.html, accessed on 6 May 2024) from 59 paired non-tumor samples and 483 lung adenocarcinoma samples, and 50 paired non-tumor samples and 486 lung squamous cell carcinoma samples (* *p* < 0.001, vs. T). (**B**) The overall survival of LUAD patients and (**C**) LUSC patients retrieved from the Kaplan–Meier Plotter database. The analysis was performed in 1161 LUAD patients and 780 LUSC patients (https://kmplot.com/analysis/, accessed on 6 May 2024). (**D**) Analysis of EZH2 protein levels by Western blot and (**E**) gene expression analysis by qPCR in lung adenocarcinoma cell lines A549, Calu3, and H2122. The expression was normalized using β-actin. LUAD, lung adenocarcinoma; LUSC, lung squamous cell carcinoma; T, tumoral; N, normal.

To evaluate *EZH2* expression in lung cancer, we accessed data from TCGA public database. We observed that EZH2 was highly expressed in LUAD and lung squamous cell carcinoma (LUSC) samples compared with normal tissues (Figure 1A). In addition, we analyzed the influence of *EZH2* expression levels on the survival of LUAD patients and observed that the overall survival of LUAD patients with high *EZH2* expression was poorer than those with low expression of *EZH2* (Figure 1B).

In vitro, we analyzed EZH2 expression in three lung adenocarcinoma cell lines A549, Calu3, and H2122. EZH2 protein expression was upregulated significantly in A549 and H2122 cell lines compared to Calu3 (Figure 1C,D), although EZH2 gene expression was higher in Calu3 and H2122 (Figure 1E). The corresponding original Western blot images for all Western blot experiments are provided in Supplementary Figures S1–S16.

### 3.2. CRISPR/Cas9-Mediated EZH2 Gene Editing Improves Differentiation and Induces Epithelial-to-Mesenchymal Transition (EMT) in the A549 Cell Line

**Figure 2:**
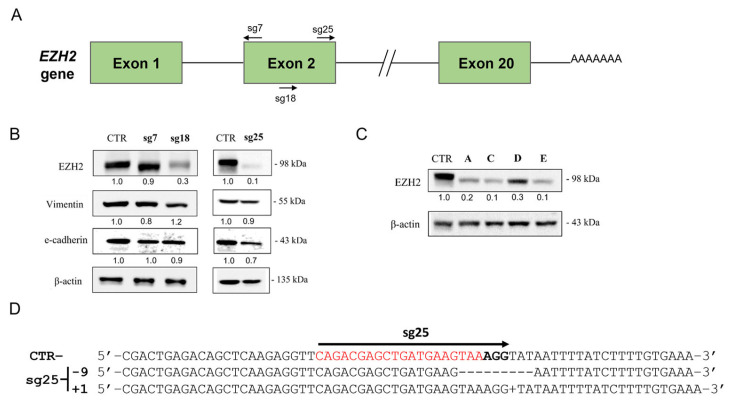
EZH2 gene editing using CRISPR/Cas9. (**A**) Schematic view of *EZH2* gene with introns and exons. The sgRNAs (sg7, sg18, and sg25) targeted the *EZH2* gene at different positions close to the second exon, near the *ATG* start codon. (**B**) EZH2, E-cadherin, and vimentin protein levels in A549-sgRNA 7, 18, and 25 compared to A549-CTR by Western blot. (**C**) EZH2 protein expression in clonal populations (Cl) ClA, ClC, ClD, and ClE derived from CRISPR/Cas9-edited A549-sg25 cells. Expression was normalized using β-actin. (**D**) Genomic DNA sequencing of A549-sg25 after CRISPR/Cas9 editing of *EZH2* gene. sgRNA target regions are highlighted in red, and PAM sequence is in bold. dsDNA repair resulted in a 9 nt deletion and 1 nt addition to the *EZH2* gene in A549-sg25. Sg, single guide; CTR, control; cl, clone; PAM, protospacer adjacent motif; dsDNA, double-strand break DNA.

Next, we used CRISPR/Cas9 to edit the *EZH2* gene and disrupt EZH2 expression in the A549 cell line. *EZH2* is a 20-exons gene located at chromosome 7 (7q36.1) and the editing targeted the second exon that contains the start codon (ATG) by guiding Cas9 with different sgRNAs (sg7, sg18, and sg25) (Figure 2A). Analysis of EZH2 protein levels in A549-edited mixed populations showed that all three sgRNAs resulted in EZH2 protein reduction; however, sg25 was the most efficient sgRNA (reduction of 80%) (Figure 2B).

Then, we derived stable clones from an A549-sg25 mixed population using limiting dilution. After clone expansion from A549-sg25 (ClA, C, D, and E), we analyzed EZH2 protein expression by Western blot. The sg25 clones showed a reduction in EZH2, but it was not stronger than the reduction observed in the mixed population of A549-sg25 (Figure 2C). In this sense, we chose to continue functional assays using the mixed population of A549-sg25. 

Next, we confirmed that CRISPR/Cas9+sgRNA25 specifically targeted the *EZH2* gene in A549-sg25 by sequencing the genomic DNA (Figure 2D and Supplementary Figure S17). The SeqScreener Gene Edit Confirmation App analysis showed successful *EZH2* gene editing with an overall efficiency of ~96% in A549 and the top indel sequences showed deletion of 9 nt (40.98%) and insertion of 1 nt (36.62%), which resulted in nonsense alterations in the protein (Supplementary Table S4 and Supplementary Figure S17). In addition, we validated that CRISPR/Cas9 did not edit the coding genes *WDR7* and *DCLK3,* two potential off-targets for sgRNA25 (Supplementary Figure S18 and Table S5).

**Figure 3:**
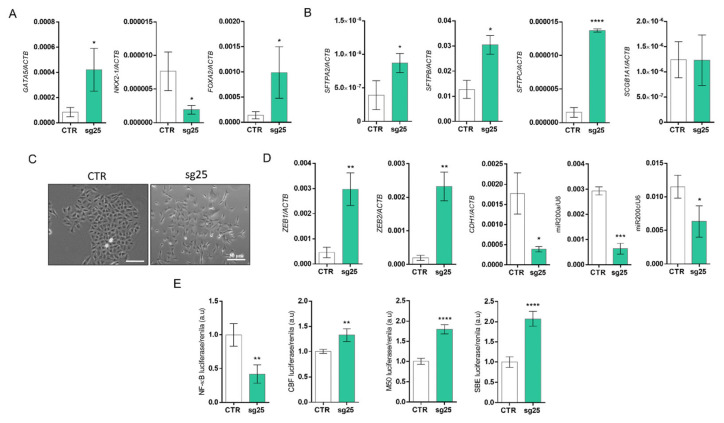
CRISPR/Cas9 targeting of the *EZH2* gene improves differentiation and induces epithelial–mesenchymal transition in lung cancer cells. (**A**) RT-qPCR analysis of gene expression of the transcription factors *NKX2-1*, *GATA5*, and *FOXA2* and (**B**) lung surfactant genes *SFTPA2*, *SFTPB*, *SFTPC,* and *SCGB1A1*. *ACTB* was used as an endogenous control. (**C**) Photomicrograph showing morphology of A549-CTR and A549-sg25 cells in culture. Magnification 100×. Scale bar: 30 μm. (**D**) Gene expression analysis of mesenchymal transcription factors *ZEB1/2* and the epithelial gene *CDH1*. *RPL19* was used as an endogenous control. RT-qPCR analysis of miR200a (left) and miR200c (41) gene expression. *RNU6B* was used as an endogenous control. (**E**) Luciferase reporter gene assay to evaluate the activity of the NF-κB, Notch, Wnt/β-catenin, and TGF-β signaling pathways. Luciferase activity was normalized using renilla activity. Data are expressed as mean ± SD (*n* = 3) for gene expression. a.u., arbitrary units; *, *p* < 0.05; **, *p* < 0.01; ***, *p* < 0.001; ****, *p* < 0.0001 vs. A549-CTR.

After validating gene editing, we investigated the effects of *EZH2* editing on lung cancer biology. CRISPR/Cas9-mediated *EZH2* gene editing significantly upregulated the expression of the transcription factors *NKX2-1*, *GATA5,* and *FOXA2*, and the lung surfactant genes *SFTPC*, *SFTPB*, *SFTPA2,* and *SCGB1A1* (Figure 3A). These combined results indicate an improvement in cell differentiation.

We also evaluated whether *EZH2* gene editing with CRISPR/Cas9 could modulate EMT. Morphologically, we observed changes in cell organization that led us to investigate the EMT process. Interestingly, when compared with the A549-CTR cell line, A549-sg25 (EZH2-edited cells) formed unpacked colonies with elongated and fusiform cell morphology typically associated with mesenchymal states (Figure 3B). This result is consistent with the increased expression of mesenchymal markers *ZEB1/2*, as well as the reduction in miR-200a/c and E-cadherin (*CDH1*) that can be observed in EZH2-edited cells (Figure 3C). The protein expression of E-cadherin also decreased in Western blot analysis; however, the expression of vimentin was not modulated (Figure 2B).

Finally, we used luciferase reporter plasmids to assess the activity of the NF-κB, Notch, Wnt/β-catenin, and TGFβ signaling pathways. Although we observed a substantial repression in NF-κB signaling in A549-sg25 compared to A549-CTR, Notch, Wnt/β-catenin, and TGFβ pathways showed increased activation in *EZH2*-edited cells, potentially contributing to the emergence of a mesenchymal phenotype in EZH2-edited cells (Figure 3D).

### 3.3. CRISPR/Cas9-Mediated EZH2 Gene Editing Has an Antitumoral Effect In Vitro in the A549 Cell Line

**Figure 4:**
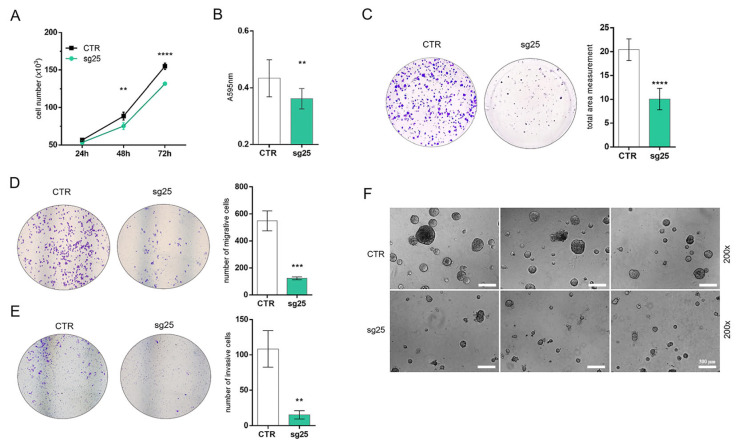
CRISPR/Cas9-mediated *EZH2* gene editing reduces the proliferation, migration, and invasion of A549 cells. (**A**) A549-sg25 cell counts were assessed at 24h, 48h, and 72 h post-seeding, based on two independent experiments performed in triplicate. (**B**) MTT assay of A549-sg25. Absorbance was measured at 595 nm. (**C**) Colony formation assay of A549-CTR compared to A549-sg25. The graph shows quantification from the colonial area. The results are representative of two independent experiments performed in triplicate. (**D**) Cell migration assay in Transwell^®^ chambers in A549-CTR cells compared to A549-sg25. Representative images of migratory cells. Data shown are representative of two independent experiments conducted in triplicate. (**E**) Cell invasion assay using Transwell^®^ chambers covered with GELTREX^®^ in A549-CTR cells compared to A549-sg25. Quantification of invasive cells in four random areas at 100× magnification. (**F**) Photomicrograph of A549-CTR and A549-sg25 cells seeded on poly (2-hydroxyethyl methacrylate) (poly-HEMA)-coated plates for suspension culture preparation. Images were taken at 200× magnification. Scale bar: 300 µm. **, *p* < 0.01; ***, *p* < 0.001; ****, *p* < 0.0001 vs. A549-CTR.

We next assessed the functional effects of EZH2 loss of expression on cell count, MTT, cell migration, invasion, and colony formation in vitro. We observed that A549-sg25 cells showed a significant reduction in cell counting after 72 h (Figure 4A), an effect accompanied by a significant reduction in cell viability of A549-sg25 analyzed by the MTT assay (Figure 4B). The colony formation assay revealed that A549-sg25 form significantly fewer colonies than A549-CTR (Figure 4C). Moreover, EZH2-edited cells also showed reduced cell migration and invasion using a Transwell^®^ insert (Figure 4D,E) and reduced capacity to form tumor spheroids in adhesion-independent growth in suspension culture (Figure 4F). These findings suggest that EZH2 suppression has an antitumoral effect in vitro, in conjunction with the improvement in cell differentiation observed in gene expression analysis.

### 3.4. CRISPR/Cas9-Mediated EZH2 Gene Editing Induces the Expression of EZH1 and Other PcG Genes

**Figure 5:**
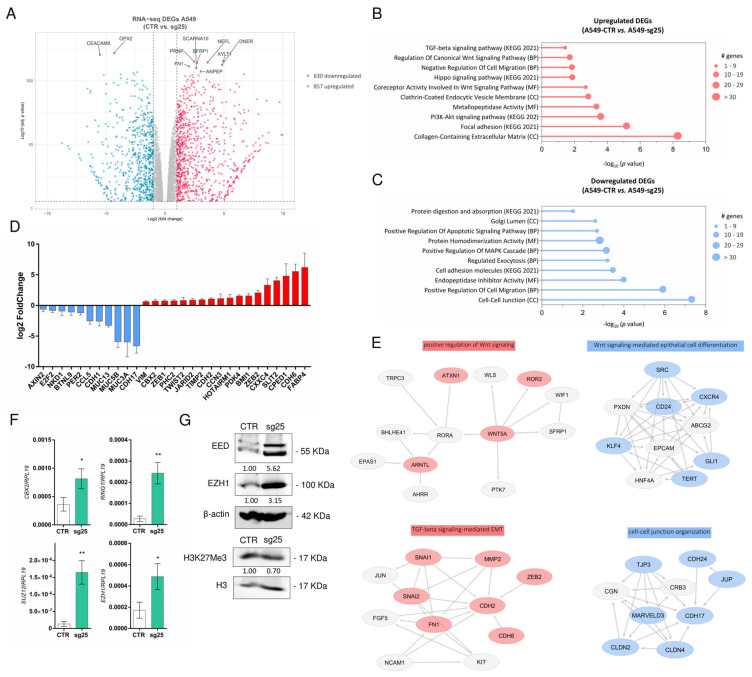
CRISPR/Cas9 targeting of the *EZH2* gene induces expression of *EZH1* and other PRC1 genes. (**A**) Volcano plot showing 630 downregulated and 857 upregulated DEGs selected to proceed with functional gene annotation (Enrichr). (**B**,**C**) Gene ontology analysis of differentially expressed genes (DEGs) in A549-sg25 cells. (**D**) Fold change in expression of epithelial and mesenchymal markers, and PRC1/2 constituent genes. (**E**) Functional enrichment of upregulated DEGs (red) and downregulated DEGs (blue) showing a protein–protein interactions network. Arrows represent hierarchical regulatory relationships between nodes, indicating up-stream or downstream interactions (**F**) Gene expression analysis of genes constituting PRC1/2 complexes. Gene expression was normalized using *RPL19*. (**G**) Western blot of EED, EZH1, and H3K27Me3 in A549-sg25 compared with A549-CTR cells. Expression was normalized using β-actin. Data are expressed as mean ± SD (*n* = 3); *, *p* < 0.05; **, *p* < 0.01 vs. A549-CTR. BP, biological process; CC, cellular component; MF, molecular function; KEGG, Kyoto Encyclopedia of Genes and Genomes. Log_2_ (fold-change) value: ±1.

To depict a broader view of EZH2-regulated genes in response to *EZH2* gene editing, we performed RNA sequencing in A549-sg25 compared with A549-CTR to identify differentially expressed genes (DEGs). We identified 630 downregulated and 857 upregulated DEGs (Figure 5A–E, Supplementary Table S7) based on the Log_2_ (fold-change) value: ±1 and performed functional enrichment analysis using online tools. RNA-seq transcriptome analysis of the most differentially expressed genes revealed increased expression of mesenchymal markers *VIM*, *ZEB1*, *ZEB2*, *TWIST*, *CDH2*, and *CDH6* and reduction in the expression of epithelial cadherin (*CDH1*/E-cadherin). An increase in the expression of genes that constitute the PRC1/2 complexes was also observed, such as *CBX2*, *BMI1*, *JARID2*, and *PHC2*. Concurrently, tumor suppressor genes *TIMP2*, *PDK4*, *CPED1*, *FABP4,* and *CXXC4* were increased, while the oncogenes *CDH17*, *MUC5B*, *MUC3A*, *MUC13*, *CCL5* and *E2F2* were reduced (Figure 5D).

Gene enrichment analysis revealed that upregulated genes were related to key processes in extracellular matrix organization, focal adhesion, the TGFβ signaling pathway, canonical Wnt signaling pathway, and PI3K/Akt signaling pathway (Figure 5B), corroborating with our luciferase reporter assays (Figure 3E), while downregulated genes were related to the apoptotic signaling pathway, regulation of MAPK cascade, cell–cell junction, endopeptidase inhibitor activity, and regulation of cell migration (Figure 5C). Protein–protein interaction network analysis with the STRING database showed that upregulated networks induce EMT and are related to increased cell proliferation and survival (Wnt signaling, TGFβ signaling, and extracellular matrix organization). On the other hand, downregulated networks are related to Wnt signaling-mediated epithelial cell differentiation, cell–cell junction organization, and embryo development (Figure 5E).

Indeed, RNA-Seq analysis pointed to a compensatory effect of PRC2/PRC1 activation in response to *EZH2* gene editing. To investigate the cooperative role between the PRC1 and PRC2 complexes, we evaluated the expression of some components of these two complexes in the A549-sg25 cell line in vitro. We observed that *EZH2* gene editing resulted in increased expression of *CBX2* and *RING1* (PRC1 components), and *SUZ12* and *EZH1* (PRC2 components) (Figure 5F). Furthermore, we observed increased protein expression of *EZH1* and *EED*, both components of PRC2, while H3K27Me3 reposition remained partially inhibited in A549-sg25 cells (Figure 5G), indicating a compensatory reactivation of non-canonical PRC2 in response to EZH2 editing.

### 3.5. CRISPR/Cas9-Mediated EZH2 Gene Editing Induces Tumor Progression In Vivo in the A549 Cell Line

**Figure 6:**
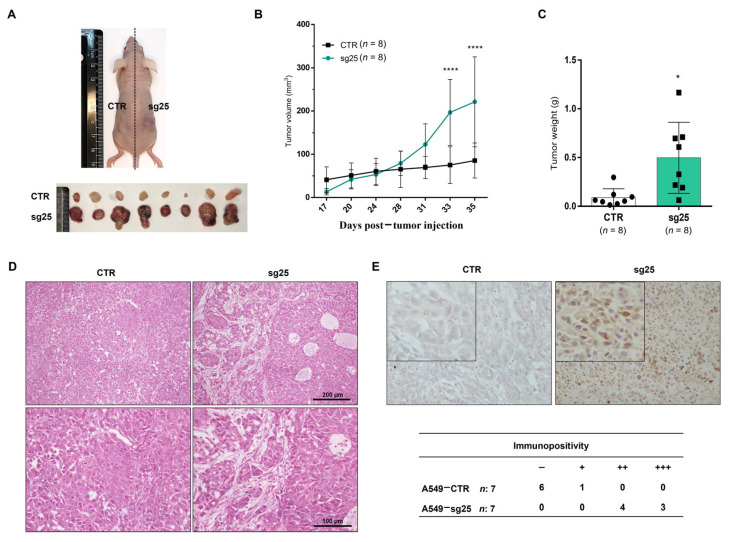
Targeted *EZH2* gene editing via CRISPR/Cas9 induces tumor progression in vivo. (**A**) Representative image of a mouse injected with A549-CTR cells (in the left flank) and A549-sg25 cells (in the right flank) and aligned tumors of 8 animals (*n* = 8). (**B**) Tumor volume progression from day 17 and day 35 (euthanasia). (**C**) Final tumor weight from eight animals. (**D**) Histology of A549-CTR tumor and A549-sg25 tumor stained with H&E. Magnification: 100× and 200×. Scale bar: 60 µm. (**E**) Immunohistochemical detection of beta-catenin in A549-derived tumors. The accumulation of nuclear β-catenin in A549-sg25 compared with A549-CTR. Tumors were counterstained with Harris hematoxylin. Immunopositivity was scored according to signal intensity as negative (–), weak (+), medium (++), and strong (+++). Magnification: 200×. *, *p* < 0.05; ****, *p* < 0.0001 vs. A549-CTR.

To investigate the effect of *EZH2* gene editing on tumor growth in vivo, we injected A549-sg25 and A549-CTR cells into opposite flanks of immunodeficient nude mice. Our results showed that A549-sg25-derived tumors exhibited a substantial increase in volume compared to A549-CTR tumors, 35 days post-injection (Figure 6A–C and Supplementary Figure S19). Histological examination of tumor sections using H&E staining revealed that A549-sg25 tumors displayed enhanced vascularization, areas of edema, cell dissociation, and interspersed proteinaceous material. Conversely, control group tumors (A549-CTR) exhibited significant regions of dense connective tissue and an organoid arrangement (Figure 6D). Immunohistochemical staining showed pronounced accumulation of nuclear β-catenin in the *EZH2*-edited cells (A549-sg25) (Figure 6E). Importantly, the analysis of *EZH2* gene editing in the resulting tumor by DNA sequencing showed a shift in the prevalence of nonsense alterations detected during gene editing in vitro to an enrichment of missense alterations in the EZH2 protein in the tumors (Supplementary Table S6 and Figure S20). The most prevalent nonsense alteration in vitro, a deletion of 9 nt detected in 40,98% of sequences, is now detected only in 2,43% of sequences in the tumor, while the missense deletion of 6 nt results in the loss of 1–2 amino acids in 44.99% of sequences in the tumor.

### 3.6. Pharmacological Blockade of EZH2 Methyltransferase Activity Improves Differentiation in A549 and H2122 Cell Lines 

To investigate if the effects of blocking EZH2/PRC2 with CRISPR/Cas9 gene editing would be mimicked by pharmacological blockade of EZH2, we used the specific allosteric inhibitor of EZH2 EPZ6438 that blocks methyltransferase activity to treat human lung adenocarcinoma cell lines A549 and H2122.

**Figure 7:**
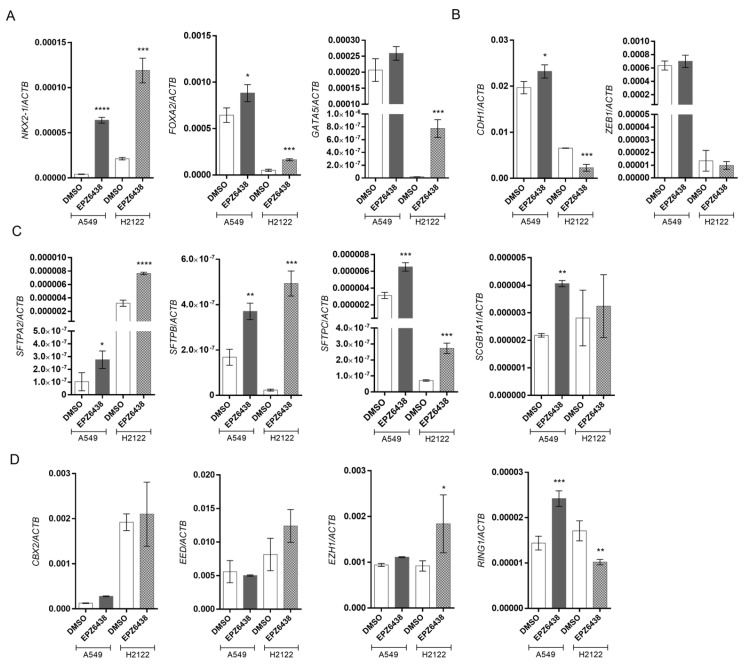
Pharmacological blockades of *EZH2* activity induce differentiation into the A549 and H2122 cell lines. (**A**) RT-qPCR analysis of gene expression of transcription factors *NKX2-1*, *GATA5*, and *FOXA2*. (**B**) Gene expression analysis of mesenchymal transcription factors *ZEB1/2* and the epithelial gene *CDH1.* (**C**) Genes encoding lung surfactants *SFTPA2*, *SFTPB*, *SFTPC*, and *SCGB1A1*. (**D**) Gene expression analysis of genes constituting PRC1/2 complexes. Gene expressions were normalized using *ACTB*. A549 and H2122 cells were treated with 5.0 µM EPZ6438 for 6 days. DMSO was used as control. Data are expressed as mean ± SD (*n* = 3); *, *p* < 0.05; **, *p* < 0.01; ***, *p* < 0.001; ****, *p* < 0.0001 vs. DSMO.

Treatment of A549 and H2122 with EPZ6438 for 6 days upregulated the expression of the transcription factors *NKX2-1*, *GATA5*, and *FOXA2* (Figure 7A) and induced the expression of the lung surfactant genes *SFTPA2*, *SFTPB*, *SFTPC,* and *SCGB1A1* (Figure 7C). Regarding EMT regulatory genes, treatment with EPZ6438 induced the expression of *CDH1* (E-cadherin) in A549 cells, while it was reduced in H2122 cells. The transcription factor *ZEB1* exhibited no significant changes in either cell line (Figure 7B). Treatment with the pharmacological inhibitor caused an increase in *RING1* expression, while changes in *CBX2* (PRC1 components), *EED,* and *EZH1* (PRC2 components) were not significant in A549 cells. In the H2122 cell line, an increase in *EZH1* and a reduction in *RING1* were observed, while *CBX2* and *EED* showed no significant changes (Figure 7D).

Our results indicate that short-term pharmacological inhibition of EZH2 activity induces redifferentiation in human lung adenocarcinoma cells, like CRISPR/Cas9 *EZH2*-edited cells, while promoting fluctuations in the expression of PcG genes.

### 3.7. Pharmacological Inhibition of EED Improves Differentiation in A549 Cells

The interaction of EED with the product of PRC2 catalysis is essential to maintain the stability of the PRC2 complex and H3K27me3 levels. The core PRC2 complex is composed of three subunits: catalytic subunit EZH2 or EZH1, scaffolding subunit SUZ12, and EED, which allosterically activates EZH2 upon binding to H3K27me3. MAK683, an EED inhibitor, binds to the domain of EED, which interacts with H3K27me3, thereby disrupting the interaction with the PRC2 complex and impairing the HMTase activity of EZH2/EZH1.

**Figure 8:**
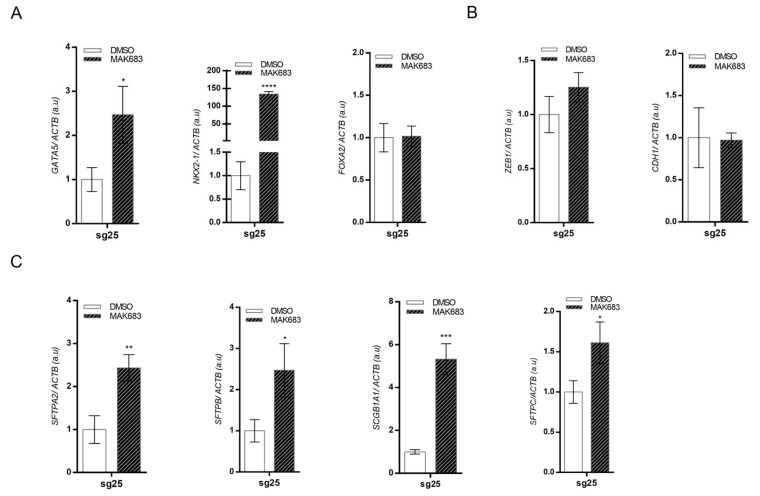
Pharmacological inhibition of *EED* activity induces differentiation in A549 EZH2-edited cells (A549-sg25). (**A**) RT-qPCR analysis of gene expression of transcription factors *GATA5*, *NKX2-1,* and *FOXA2*. (**B**) Gene expression analysis of mesenchymal transcription factor *ZEB1* and the epithelial gene *CDH1.* (**C**) Genes encoding lung surfactants *SFTPA2*, *SFTPB*, *SFTPC,* and *SCGB1A1*. Gene expressions were normalized using *ACTB*. A549-sg25 cells were treated with 2.0 µM MAK683 for 6 days. DMSO was used as control. Data are expressed as mean ± SD (*n* = 3); *, *p* < 0.05; **, *p* < 0.01; ***, *p* < 0.001; ****, *p* < 0.0001 vs. DMSO.

To investigate the effect of EED blockage in EZH2-edited cells (A549-sg25) that show reactivation of EZH1 and EED, we used the MAK683 inhibitor. Treatment of A549-sg25 with MAK683 for 6 days strongly upregulated the expression of the transcription factor *NKX2-1,* with an additive effect in *GATA5* (Figure 8A). Moreover, EED inhibition in EZH2-edited cells (A549-sg25) resulted in upregulation of *SFTPA2*, *SFTPB,* and *SCGB1A*, genes that were already induced by EZH2 disruption by CRISPR/Cas9, but no reversal in the EMT TF *ZEB1* induction or in *CDH1* inhibition was observed in EZH2-edited cells (Figure 8B,C).

## 4. Discussion

Increased EZH2 levels have emerged as a mark of advanced and metastatic disease, promoting proliferation, mobility, and invasion in many solid tumors [24,25,26,27,28,29]. In this context, the reactivation of EZH2/PRC2 in lung cancer could contribute to the development of aggressive characteristics detected at diagnosis of lung cancer, such as frequent metastatic dissemination, which reduces patient survival. Thus, in this study, we evaluated the role of EZH2 in lung cancer biology by blocking EZH2 activity. We applied CRISPR/Cas9-mediated gene editing to precisely silence EZH2 expression in the A549 cell line, derived from human lung adenocarcinoma. As consequence, the functional assays showed that *EZH2* gene editing impaired cell proliferation, migration, invasion, and suspension growth of A549 cells, and also reduced the signaling transduction of the nuclear factor kappa B (NF-κB) pathway, a pro-inflammatory transcription factor that contributes to tumor progression through its impact on cellular senescence, apoptosis, stress responses, and tumorigenesis [30]. Indeed, EZH2 contributes to oncogenesis not only through its classical role in histone H3 lysine 27 methylation, but also by directly interacting with transcription factors and oncogenes to modulate gene expression [31]. One notable non-histone function of EZH2 is its ability to methylate Gata4 at lysine 299, attenuating its transcriptional activity and affecting cardiac development [32]. In the context of breast cancer, EZH2 physically associates with RelA and RelB, enhancing the transcription of NF-κB target genes independently of its histone methyltransferase activity [33].

The activation of the PRC2/EZH2 complex in lung cancer would act to repress tumor suppressor genes, pathways, and lung cell differentiation, enhancing the oncogenic process. The analysis of lung differentiation genes expression showed that EZH2 inhibition (either by CRISPR/Cas9 or EZH2 inhibitor EPZ6438) induced the expression of transcription factors *GATA5* and *FOXA2*, and the lung surfactants *SFTPA2*, *SFTPB*, *SFTPC*, and *SCGB1A1* in A549 cells. Transcription factors contribute to a wide range of important cellular physiological and pathological processes, regulating the transcription of a series of target genes that control lung cell growth, proliferation, and differentiation. Indeed, the potential tumor suppressor action of *GATA4/5* and *FOXA2* has been demonstrated in lung cancer [34,35,36,37,38].

Pulmonary surfactants are surface-active substances composed mainly of lipids and proteins contained within the alveolar lining fluid of the lungs. They play a key role in preventing alveolar collapse and ensuring bronchiolar patency during normal breathing [39]. However, their expression is decreased in lung adenocarcinoma [40]. Studies have indicated that the surfactants *SFTPA* and *SFTPD* negatively regulate epidermal growth factor (EGF) signaling, thereby inhibiting cell proliferation, migration, and invasion in A549 and H441 cells [40,41,42]. Downregulation of *SFTPB* activates the Akt pathway, leading to the progression of NSCLC [43]. Moreover, inactivating mutations in the *SFTPA* gene are associated with tumor progression and smoking history [44], and altogether suggest that lung surfactants function as tumor suppressor genes and prognostic biomarkers in lung cancer [45]. Collectively, our results suggest that *EZH2* gene editing elicits an antitumoral effect in vitro by modulating the network of transcription factors, surfactants and signaling pathways, reinforcing the oncogenic role of EZH2 activation in lung cancer, which corroborates with studies in other types of cancer.

On the other hand, intriguingly, *EZH2* gene editing also elicited pro-tumoral effects, such the activation of Wnt/β-catenin and TGFβ oncogenic pathways, linked to alterations in cell morphology that suggest a shift to a more mesenchymal phenotype. Epithelial–mesenchymal transition (EMT) is a reversible and crucial process in cancer progression, which allows cells to become invasive, metastatic, and capable of reverting to an epithelial state in a process known as mesenchymal–epithelial transition (MET) [46]. This phenomenon is controlled by transcription factors (TWIST1/2, SNAI1/2, and ZEB1/2), which coordinate the repression of genes that maintain the epithelial state and the activation of genes that induce mesenchymal characteristics [47]. MET, in turn, is associated with a colonization of new tissues by carcer cells in metastatic sites [3].

However, cells that activate EMT programs in adult tissues under pathological conditions typically express combinations of epithelial and mesenchymal markers and rarely complete the EMT program [48]. Therefore, the mixed population of EZH2-edited cells (A549-sg25) may represent heterogeneous populations of tumor cells, each in a different state of epigenetic change or trapped in an intermediate phenotype. Our results show that, despite the antitumoral effect observed in vitro, *EZH2* gene editing in the A549 cell line resulted in alterations in the EMT process. We observed an increase in ZEB1/2 levels and increased activity of Notch, Wnt/β-catenin, and TGFβ signaling pathways in *EZH2*-edited cells. This coincides with the elongated and fusiform cell morphology typically associated with the mesenchymal phenotype.

In human lung adenocarcinoma cells, TGFβ1 enhances invasive and metastatic potential by promoting epithelial–mesenchymal transition (EMT), primarily through downregulation of E-cadherin [49]. EMT regulation by TGFβ occurs via both Smad-dependent and Smad-independent pathways, including NF-κB/Snail, PI3K/AKT, JAK/STAT3, and MAPK/ERK1/2 signaling pathways [50]. Wnt/β-catenin, when overactivated, is associated with metastasis, migration, invasion, and chemotherapy resistance in lung cancer [51], and is associated with β-catenin nuclear accumulation and its binding to TCF/LEF to induce MYC and ZEB1 transcription and repress miR-200 transcription [52]. Notch activation is associated with poor prognosis in lung adenocarcinoma and correlates with an embryonic stem cell-like gene signature [53,54].

Our observations are consistent with reports demonstrating that genetic deletion of *EZH2* promotes cancer cell residency in the mesenchymal state during the reversible epithelial–mesenchymal transition, facilitating tumor colonization in vivo in lung cancer [55,56]. Thus, the activation of the Wnt/β-catenin, Notch, and TGF-β signaling pathways potentially contributed to the emergence of a mesenchymal phenotype in our cultured cells that could impact tumor growth in vivo. In this context, we also analyzed the effects of *EZH2* gene editing on tumor formation in vivo by injecting cells into immunodeficient mice. We observed that the edited cells (A549-sg25) formed larger tumors that showed induction of β-catenin expression in vivo by IHC, corroborating the activation of β-catenin signaling in the luciferase reporter assay in vitro, and indicating the participation of this pathway in response to *EZH2* blockage. Indeed, the investigation of EZH2 blockage effects in glioblastoma showed that *EZH2* knockdown resulted in an early effect of reduction in the ability for cell proliferation and increased survival; however, prolonged inhibition of EZH2 led to increased cell proliferation, redirecting the cells to an undifferentiated stem cell state and resulting in tumor progression [57]. Interestingly, in our study, the endpoint tumor formed by *EZH2*-edited cells (A549-sg25 cells) after 35 days of injection revealed a distinct *EZH2* gene editing indel signature compared to its in vitro counterpart in the sequencing, potentially influenced by prolonged growth in vivo. We recognize that the cellular heterogeneity from the A549sg25 mixed population contributed to the in vivo uprising of different clones that outgrew and formed tumors. This is demonstrated by the decrease in frequency of nonsense mutations in the *EZH2* gene that were prevalent in vitro prior to injection into nude mice, and the increase in prevalence of rare clones with missense mutations after tumor growth.

Moreover, to elucidate whether the cooperative effect between the PRC1 and PRC2 complexes was associated with A549-sg25 tumor growth in vivo, we evaluated the expression of some PRC1 and PRC2 components in vitro. We observed that gene editing of *EZH2* resulted in increased expression of *CBX2* and *RING1*, two components of PRC1, and an increase in *EED*, *EZH1*, and *SUZ12* (PRC2 components). Furthermore, we observed increased protein levels of EZH1 and EED, indicating a compensatory reactivation of non-canonical PRC2 effects in response to *EZH2* editing, as previously described in embryonic stem cells [15]. However, interestingly, our data show that H3K27Me3 remains partially inhibited in EZH2-edited cells. Collectively, our data reinforces the hypothesis of PRC1 and PRC2 upregulation in response to EZH2 gene editing and indicates that EZH1 and EED reactivation may also act through a noncanonical pathway to promote tumorigenesis in vivo.

There are two homologous sequences of EZH: the paralogs *EZH2* and *EZH1*. *EZH1* is part of a PRC2 complex like the one containing *EZH2*, and they share an overlapping set of target genes [58]. Although EZH2 was initially identified as the sole histone methyltransferase of PRC2, recent studies have shown that EZH1 is part of a noncanonical PRC2 complex that catalyzes the methylation of H3K27 and prevents the derepressing of PRC2 target genes [16].

The overlap of PcG target genes between PRC1, PRC2, and H3K27me2/3 has been previously described [51,59,60]. Unlike invertebrates, vertebrates have several paralogs of most PcG genes [61] and, although the *EZH2* gene is well characterized, the function of its homologous gene *EZH1* remains largely unknown. The RNA levels of *EZH1* and *EZH2* appear to be inversely correlated, with *EZH1* being ubiquitously expressed, while *EZH2* is present only in proliferating tissues [14]. The presence of the H3K27me2/3 mark in non-proliferative tissue may be a consequence of EZH1 protection against demethylases by compacting chromatin and preventing aberrant activation of many EZH2 target genes [15]. Genomic analyses have demonstrated locus-specific persistence of H3K27me3 despite EZH2 inactivation, suggesting new DNA methylation through a compensatory mechanism exerted by EZH1 in myeloid–lymphoid leukemia [62]. Compared to EZH2 depletion, loss of EED more effectively abrogates PRC2 function, avoiding the compensatory effect exerted by EZH1 [63]. These effects include more profound changes in gene expression, with downregulation of Myc target genes and upregulation of PRC2 targets [64].

Indeed, our results on combinatorial inhibition of EED with MAK693 in *EZH2*-edited cells indicate an additive effect for some genes, such as *GATA5*, *NKX2-1*, *SFTPA2,* and *SFTPB,* but does not block EMT reactivation. Also, a previous study has shown that CBX2, a component of canonical PRC1, is upregulated in lung adenocarcinoma similarly to EZH2, and the high expression of both genes in combination is more significantly associated with poor prognosis than each gene alone. Moreover, the dual inhibition of CBX2 and EZH2 led to more significant cellular effects than the inhibition of either alone [10].

Since the discovery of EZH2/PRC2 deregulation in lung cancer, studies have tried to uncover the role of epigenetic silencing in cancer biology using different strategies. Genetically Engineered Mouse Models (GEMMs) based on Kras activation with concomitant deletion of *Ezh2* or *Eed*, in a context with or without p53 deletion, provided intriguing results when focusing on lung cancer formation (oncogenesis) in vivo [56,65,66].

In our study, we provide a different view focused on lung cancer progression and therapeutics as we modulated EZH2/PRC2 activity in an established lung adenocarcinoma cell line. Indeed, EZH2 expression is induced by MAPK signaling in KRas-mutated lung cancer lines [67], correlating with KRAS expression in human lung adenocarcinoma [68]. In this context, we hypothesize that EZH2 is induced mainly by the Ras oncogene, not excluding the existence of other mechanisms, to promote tumor progression. Our model cell line A549 is widely used in lung cancer studies and harbors the KRas^G12S^ and STK11^Q37*^ mutations but is wild-type for *Tp53*. We reckon that our findings for gene editing may be limited to *TP53* wild-type cancer, and we reinforce that *TP53* inactivating mutations are prevalent in lung adenocarcinoma (~46%) and often correlate with KRas mutations (~33%) [6]. However, our data show a similar effect for EZH2 inhibition with EPZ6438 in the H2122 cell line (KRAS^G12C^, TP53^Q16L,C176F^) and A549, indicating that EZH2/PRC2-mediated epigenetic repression regulates lung differentiation irrespective of *TP53* status.

## 5. Conclusions

The findings of our study show that targeting *EZH2* in lung adenocarcinoma may be challenging as CRISPR/Cas9 may edit the *EZH2* gene in a heterogeneous fashion, creating a mixed population of edited cancer cells, which ultimately may lead to a tumor growing in vivo due to clonal selection. Moreover, although blocking PRC2/EZH2 function with either CRISPR/Cas9 or EPZ6438/MAK693 results in some extent of lung pro-differentiation induction, there is also an induction of EMT that could be a source of new metastatic dissemination if any of these approaches are implemented for human adenocarcinoma patients’ treatment. Thus, combining EZH2 inhibition with other interventions to prevent PRC2/PRC1 reactivation may help improve the applicability of epigenetic blockage as a potential therapy for lung cancer.

## Data Availability

The RNAseq data presented in this study are available as Supplementary Materials, as described in the Section 2 (Table S7).

## Supplementary Materials

The following supporting information can be downloaded at: https://www.mdpi.com/article/10.3390/biology15030251/s1, Figures S1–S16: Full-length WB, uncropped figures; Figure S17: EZH2 gene editing with CRISPR/Cas9; Figure S18: Off-target sequencing; Figure S19: Mice weight monitoring and endpoint image after tumor xenotransplant; Figure S20: Alignment of amino acid changes after EZH2 gene editing with sgRNA25; Table S1: Lung cancer cell lines and culture conditions; Table S2: List of primers used in this study; Table S3: Validation of CRISPR/Cas9-mediated EZH2 gene editing with sgRNA25 in A549 cells in vitro using the SeqScreener Gene App; Table S4: List of antibodies and dilutions for protein expression analysis; Table S5: List of potential off-target genes of sgRNA25; Table S6: Validation of CRISPR/Cas9-mediated EZH2 gene editing with sgRNA25 in A549 tumor in vivo using the SeqScreener Gene App; Table S7: Differentially expressed genes (A549 CRISPR EZH2 sg25 /A549 empty) in RNA-Seq.

## Abbreviations

The following abbreviations are used in this manuscript:

BP	Biological Process
CC	Cellular Component
DEGs	Differentially Expressed Genes
EGF	Epidermal Growth Factor
EMT	Epithelial–Mesenchymal Transition
EZH2	Enhancer of Zest Homolog 2
FPKM	Fragments Per Kilobase of transcript per Million mapped reads
H&E	Hematoxylin and Eosin
IHC	Immunohistochemical
KEGG	Kyoto Encyclopedia of Genes and Genomes
LUAD	Lung adenocarcinoma
LUSC	Lung squamous cell carcinoma
MF	Molecular Function
NF-κB	Nuclear Factor kappa B
NSCLC	Non-small Cell Lung Cancer
PcG	Polycomb Group
PRC2	Polycomb Repressive Complex 2
TCGA	The Cancer Genome Atlas
TPM	Transcripts Per Million
