# Supplementary material for: Dual Effect of EZH2 Gene Editing with CRISPR/Cas9 in Lung Cancer
Source: Biology (Basel). 2026 Jan 29;15(3):251. doi: 10.3390/biology15030251 (PMC12896556; doi:10.3390/biology15030251)
Supplement: Supplementary file 1 [file biology-15-00251-s001.zip › Supplementary Figures S1-S16 WB uncropped.pdf]

Supplementary Materials

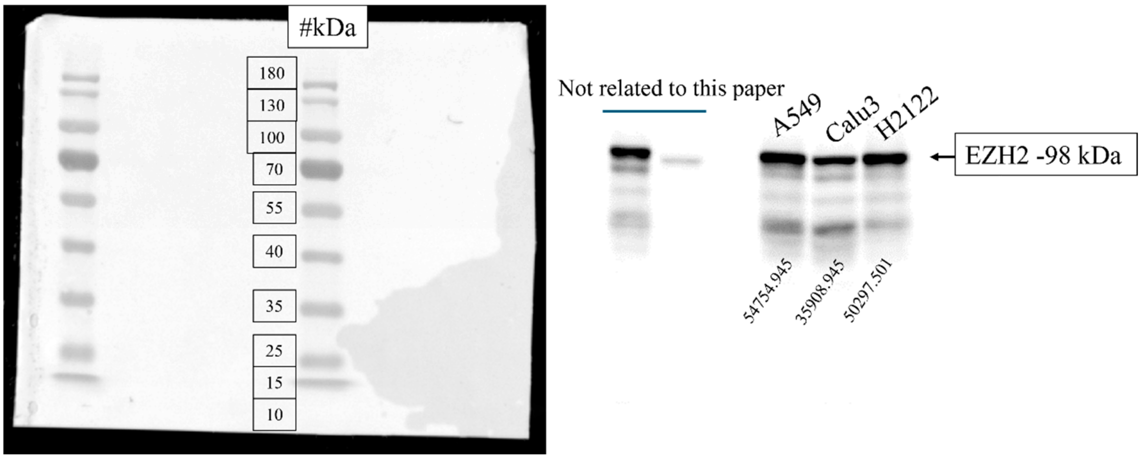

Figure S1. Western blot membrane of EZH2 (~98 kDa) protein detected with rabbit anti-EZH2 (D2C9; 1:1000; Cell signaling, Massachusetts, EUA) antibody diluted with 5% nonfat dry milk in Tris-buffered saline 0.1% Tween-20. Gel-separated proteins were transferred to nitrocellulose Hybond-ECL membranes (0.45 µm pore size; Amersham Biosciences, Little Chalfont, UK) by semi dry electroblotting at 30 V, overnight. Membrane was incubated with a horseradish peroxidase-conjugated secondary antibody (RPN1231-2ML; 1:4000; GE Healthcare, Little Chalfont, UK) and developed with luminol and p-coumaric acid (Sigma) reagents in the presence of hydrogen peroxide. #Weight marker (molecular weight in kDa): Thermo Scientific™/PageRuler™ Prestained Protein Ladder, 10 to 180 kDa; catalogue number: 26616. The densitometry analysis was performed using the Image J program.

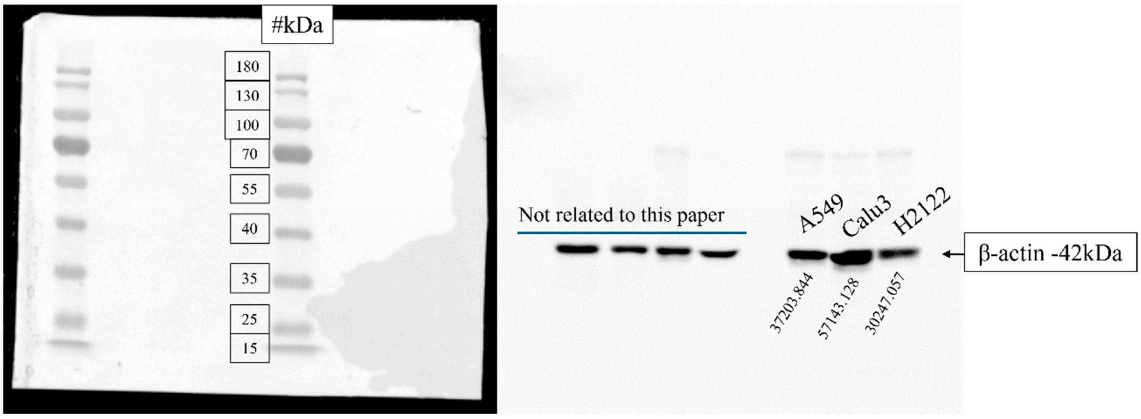

Figure S2. Western blot membrane of β-Actin (~42 kDa) protein detected with mouse anti-β-Actin (C4; 1:1000; Santa cruz, CA, USA) antibody diluted with 5% nonfat dry milk in Tris-buffered saline 0.1% Tween-20. Gel-separated proteins were transferred to nitrocellulose Hybond-ECL membranes (0.45 µm pore size; Amersham Biosciences, Little Chalfont, UK) by semi dry electroblotting at 30 V, overnight. Membrane was incubated with a horseradish peroxidase-conjugated secondary antibody (RPN1231-2ML; 1:4000; GE Healthcare, Little Chalfont, UK) and developed with luminol and p-coumaric acid (Sigma) reagents in the presence of hydrogen peroxide. #Weight marker (molecular weight in kDa): Thermo Scientific™/PageRuler™ Prestained Protein Ladder, 10 to 180 kDa; catalogue number: 26616. The densitometry analysis was performed using the Image J program.

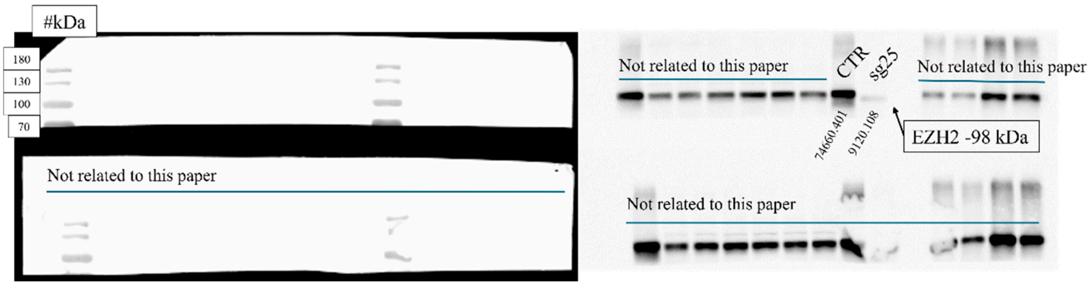

Figure S3. Western blot membrane of EZH2 (~98 kDa) protein detected with rabbit anti-EZH2 (D2C9; 1:1000; Cell signaling, Massachusetts, EUA) antibody diluted with 5% nonfat dry milk in Tris-buffered saline 0.1% Tween-20. Gel-separated proteins were transferred to nitrocellulose Hybond-ECL membranes (0.45 µm pore size; Amersham Biosciences, Little Chalfont, UK) by semi dry electroblotting at 30 V, overnight. Membrane was incubated with a horseradish peroxidase-conjugated secondary antibody (RPN1231-2ML; 1:4000; GE Healthcare, Little Chalfont, UK) and developed with luminol and p-coumaric acid (Sigma) reagents in the presence of hydrogen peroxide. #Weight marker (molecular weight in kDa): Thermo Scientific™/PageRuler™ Prestained Protein Ladder, 10 to 180 kDa; catalogue number: 26616. The densitometry analysis was performed using the Image J program.

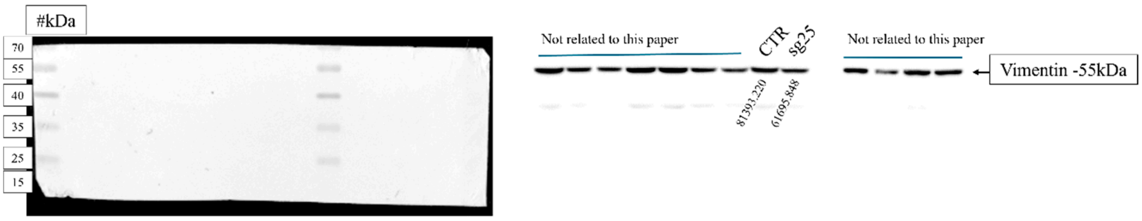

Figure S4. Western blot membrane of Vimentin (~55 kDa) protein detected with mouse anti-Vimentin (RV202; 1:1000; Santa cruz, CA, USA) antibody diluted with 5% nonfat dry milk in Tris-buffered saline 0.1% Tween-20. Gel-separated proteins were transferred to nitrocellulose Hybond-ECL membranes (0.45 µm pore size; Amersham Biosciences, Little Chalfont, UK) by semi dry electroblotting at 30 V, overnight. Membrane was incubated with a horseradish peroxidase-conjugated secondary antibody (RPN1231-2ML; 1:4000; GE Healthcare, Little Chalfont, UK) and developed with luminol and p-coumaric acid (Sigma) reagents in the presence of hydrogen peroxide. #Weight marker (molecular weight in kDa): Thermo Scientific™/PageRuler™ Prestained Protein Ladder, 10 to 180 kDa; catalogue number: 26616. The densitometry analysis was performed using the Image J program.

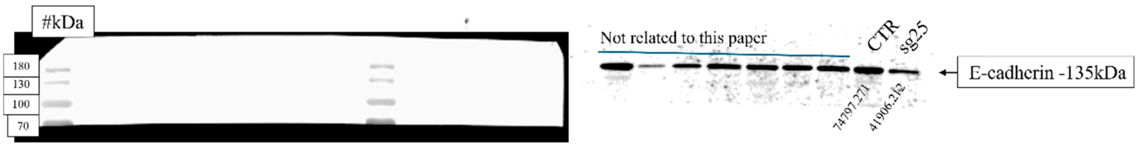

Figure S5. Western blot membrane of E-Cadherin (~135 kDa) protein detected with mouse anti-E-Cadherin (4A2; 1:1000; Cell signaling, Massachusetts, EUA) antibody diluted with 5% nonfat dry milk in Tris-buffered saline 0.1% Tween-20. Gel-separated proteins were transferred to nitrocellulose Hybond-ECL membranes (0.45 µm pore size; Amersham Biosciences, Little Chalfont, UK) by semi dry electroblotting at 30 V, overnight. Membrane was incubated with a horseradish peroxidase-conjugated secondary antibody (RPN1231-2ML; 1:4000; GE Healthcare, Little Chalfont, UK) and developed with luminol and p-coumaric acid (Sigma) reagents in the presence of hydrogen peroxide. #Weight marker (molecular weight in kDa): Thermo Scientific™/PageRuler™ Prestained Protein Ladder, 10 to 180 kDa; catalogue number: 26616. The densitometry analysis was performed using the Image J program.

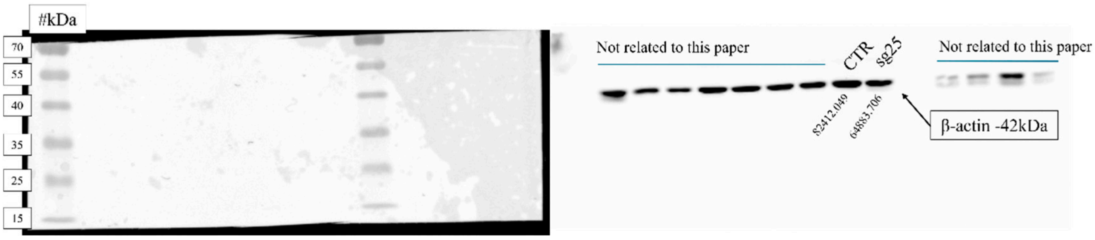

Figure S6. Western blot membrane of β-Actin (~42 kDa) protein detected with mouse anti-β-Actin (C4; 1:1000; Santa cruz, CA, USA) antibody diluted with 5% nonfat dry milk in Tris-buffered saline 0.1% Tween-20. Gel-separated proteins were transferred to nitrocellulose Hybond-ECL membranes (0.45 µm pore size; Amersham Biosciences, Little Chalfont, UK) by semi dry electroblotting at 30 V, overnight. Membrane was incubated with a horseradish peroxidase-conjugated secondary antibody (RPN1231-2ML; 1:4000; GE Healthcare, Little Chalfont, UK) and developed with luminol and p-coumaric acid (Sigma) reagents in the presence of hydrogen peroxide. #Weight marker (molecular weight in kDa): Thermo Scientific™/PageRuler™ Prestained Protein Ladder, 10 to 180 kDa; catalogue number: 26616. The densitometry analysis was performed using the Image J program.

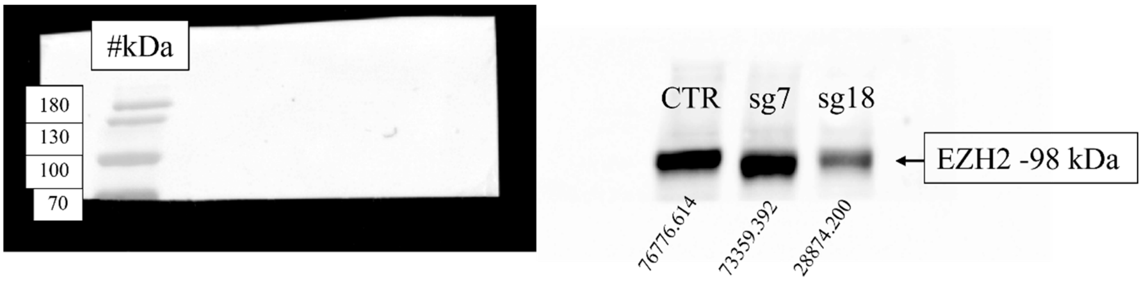

Figure S7. Western blot membrane of EZH2 (~98 kDa) protein detected with rabbit anti-EZH2 (D2C9; 1:1000; Cell signaling, Massachusetts, EUA) antibody diluted with 5% nonfat dry milk in Tris-buffered saline 0.1% Tween-20. Gel-separated proteins were transferred to nitrocellulose Hybond-ECL membranes (0.45 µm pore size; Amersham Biosciences, Little Chalfont, UK) by semi dry electroblotting at 30 V, overnight. Membrane was incubated with a horseradish peroxidase-conjugated secondary antibody (RPN1231-2ML; 1:4000; GE Healthcare, Little Chalfont, UK) and developed with luminol and p-coumaric acid (Sigma) reagents in the presence of hydrogen peroxide. #Weight marker (molecular weight in kDa): Thermo Scientific™/PageRuler™ Prestained Protein Ladder, 10 to 180 kDa; catalogue number: 26616. The densitometry analysis was performed using the Image J program.

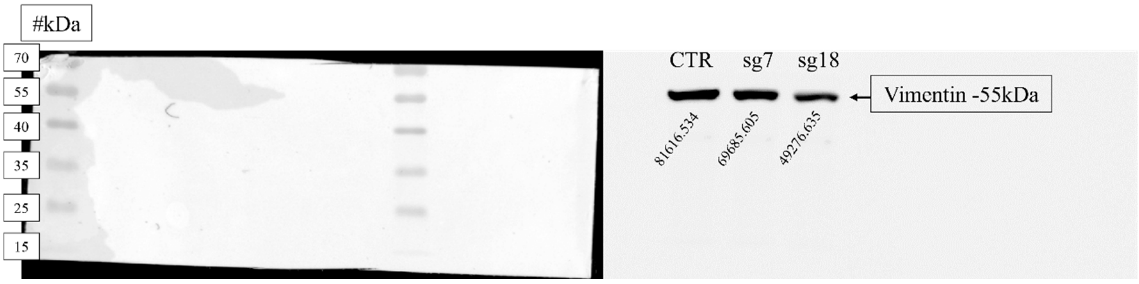

Figure S8. Western blot membrane of Vimentin (~55 kDa) protein detected with mouse anti-Vimentin (RV202; 1:1000; Santa cruz, CA, USA) antibody diluted with 5% nonfat dry milk in Tris-buffered saline 0.1% Tween-20. Gel-separated proteins were transferred to nitrocellulose Hybond-ECL membranes (0.45 µm pore size; Amersham Biosciences, Little Chalfont, UK) by semi dry electroblotting at 30 V, overnight. Membrane was incubated with a horseradish peroxidase-conjugated secondary antibody (RPN1231-2ML; 1:4000; GE Healthcare, Little Chalfont, UK) and developed with luminol and p-coumaric acid (Sigma) reagents in the presence of hydrogen peroxide. #Weight marker (molecular weight in kDa): Thermo Scientific™/PageRuler™ Prestained Protein Ladder, 10 to 180 kDa; catalogue number: 26616. The densitometry analysis was performed using the Image J program.

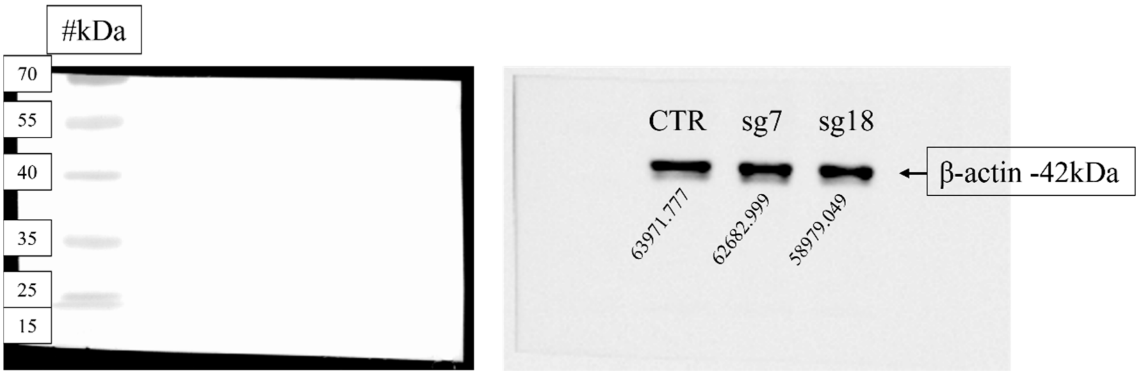

Figure S9. Western blot membrane of β-Actin (~42 kDa) protein detected with mouse anti-β-Actin (C4; 1:1000; Santa cruz, CA, USA) antibody diluted with 5% nonfat dry milk in Tris-buffered saline 0.1% Tween-20. Gel-separated proteins were transferred to nitrocellulose Hybond-ECL membranes (0.45 μm pore size; Amersham Biosciences, Little Chalfont, UK) by semi dry electroblotting at 30 V, overnight. Membrane was incubated with a horseradish peroxidase-conjugated secondary antibody (RPN1231-2ML; 1:4000; GE Healthcare, Little Chalfont, UK) and developed with luminol and p-coumaric acid (Sigma) reagents in the presence of hydrogen peroxide. #Weight marker (molecular weight in kDa): Thermo Scientific™/PageRuler™ Prestained Protein Ladder, 10 to 180 kDa; catalogue number: 26616. The densitometry analysis was performed using the Image J program.

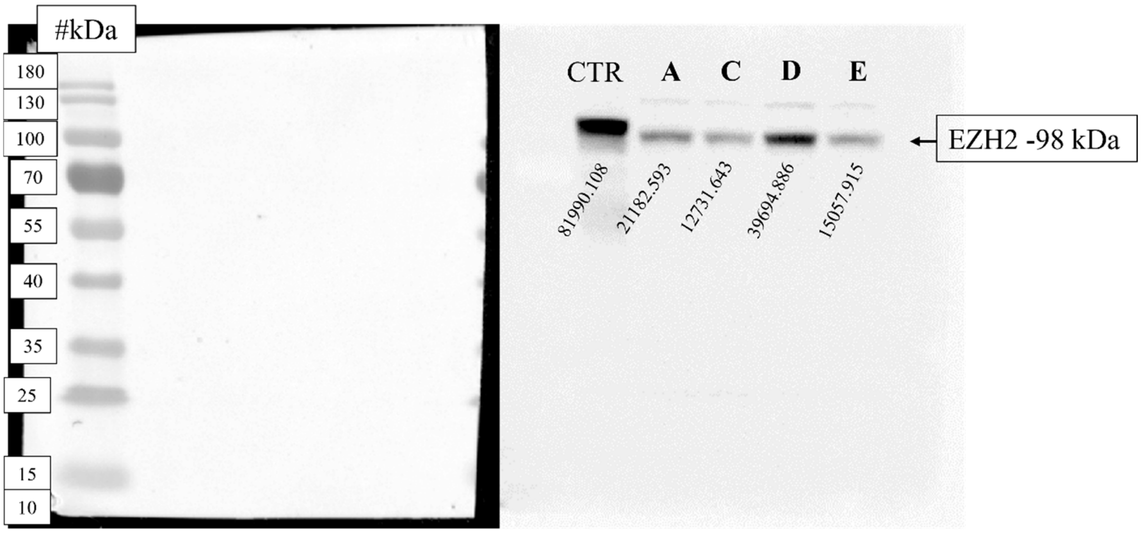

Figure S10. Western blot membrane of EZH2 (~98 kDa) protein detected with rabbit anti-EZH2 (D2C9; 1:1000; Cell signaling, Massachusetts, EUA) antibody diluted with 5% nonfat dry milk in Tris-buffered saline 0.1% Tween-20. Gel-separated proteins were transferred to nitrocellulose Hybond-ECL membranes (0.45 µm pore size; Amersham Biosciences, Little Chalfont, UK) by semi dry electroblotting at 30 V, overnight. Membrane was incubated with a horseradish peroxidase-conjugated secondary antibody (RPN1231-2ML; 1:4000; GE Healthcare, Little Chalfont, UK) and developed with luminol and p-coumaric acid (Sigma) reagents in the presence of hydrogen peroxide. #Weight marker (molecular weight in kDa): Thermo Scientific™/PageRuler™ Prestained Protein Ladder, 10 to 180 kDa; catalogue number: 26616. The densitometry analysis was performed using the Image J program.

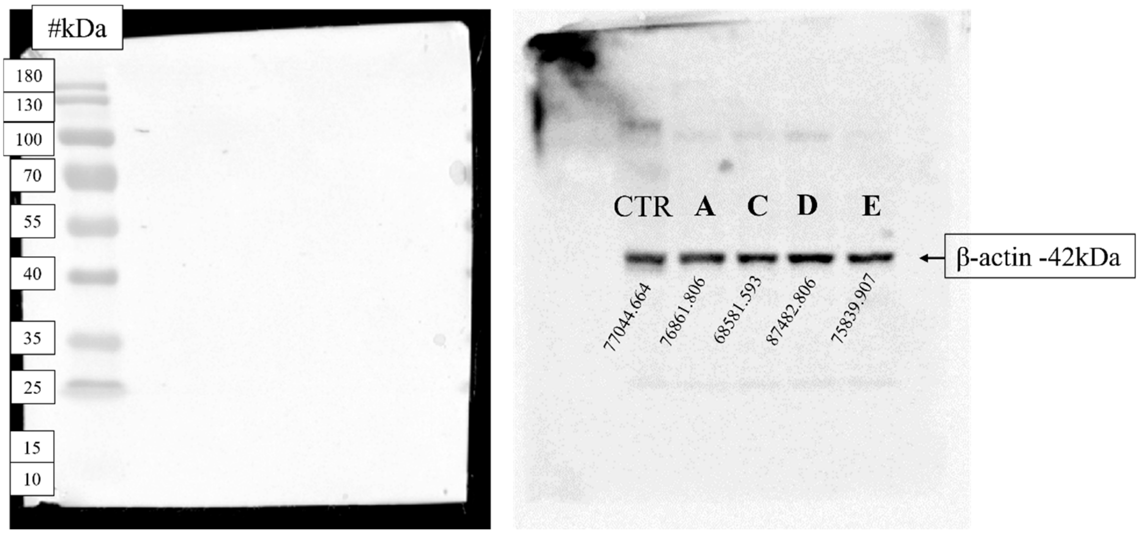

Figure S11. Western blot membrane of β-Actin (~42 kDa) protein detected with mouse anti-β-Actin (C4; 1:1000; Santa cruz, CA, USA) antibody diluted with 5% nonfat dry milk in Tris-buffered saline 0.1% Tween-20. Gel-separated proteins were transferred to nitrocellulose Hybond-ECL membranes (0.45 µm pore size; Amersham Biosciences, Little Chalfont, UK) by semi dry electroblotting at 30 V, overnight. Membrane was incubated with a horseradish peroxidase-conjugated secondary antibody (RPN1231-2ML; 1:4000; GE Healthcare, Little Chalfont, UK) and developed with luminol and p-coumaric acid (Sigma) reagents in the presence of hydrogen peroxide. #Weight marker (molecular weight in kDa): Thermo Scientific™/PageRuler™ Prestained Protein Ladder, 10 to 180 kDa; catalogue number: 26616. The densitometry analysis was performed using the Image J program.

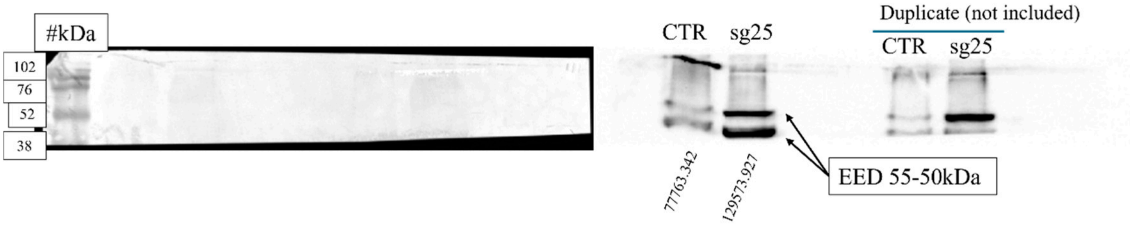

Figure S12. Western blot membrane of EED (~55-50 kDa) protein detected with rabbit anti-EED (E4L6E; 1:1000; Cell signaling, Massachusetts, EUA) antibody diluted with 5% nonfat dry milk in Tris-buffered saline 0.1% Tween-20. Gel-separated proteins were transferred to nitrocellulose Hybond-ECL membranes (0.45 µm pore size; Amersham Biosciences, Little Chalfont, UK) by semi dry electroblotting at 30 V, overnight. Membrane was incubated with a horseradish peroxidase-conjugated secondary antibody (RPN1231-2ML; 1:4000; GE Healthcare, Little Chalfont, UK) and developed with luminol and p-coumaric acid (Sigma) reagents in the presence of hydrogen peroxide. #Weight marker (molecular weight in kDa): Amersham™ ECL™ Rainbow™ Marker - Full range, 12 to 225 kDa; catalogue number: RPN800E. The densitometry analysis was performed using the Image J program.

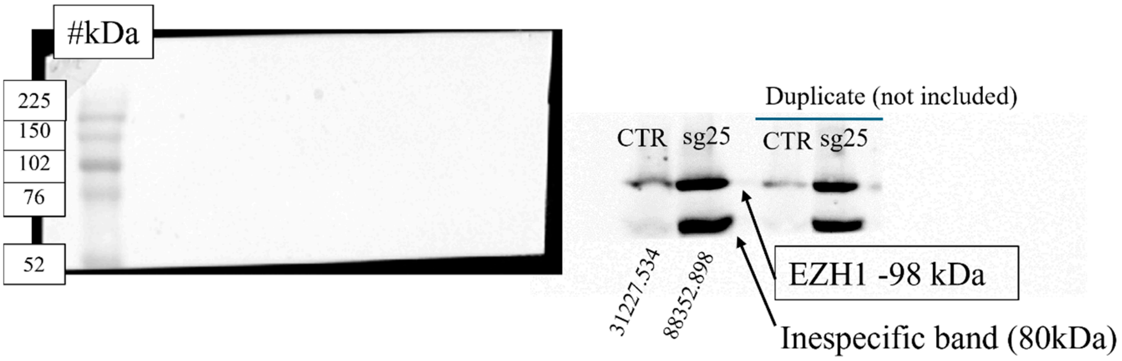

Figure S13. Western blot membrane of EZH1 (~98 kDa) protein detected with rabbit anti-EZH1 (D7D5DE; 1:1000; Cell signaling, Massachusetts, EUA) antibody diluted with 5% nonfat dry milk in Tris-buffered saline 0.1% Tween-20. Gel-separated proteins were transferred to nitrocellulose Hybond-ECL membranes (0.45 µm pore size; Amersham Biosciences, Little Chalfont, UK) by semi dry electroblotting at 30 V, overnight. Membrane was incubated with a horseradish peroxidase-conjugated secondary antibody (RPN1231-2ML; 1:4000; GE Healthcare, Little Chalfont, UK) and developed with luminol and p-coumaric acid (Sigma) reagents in the presence of hydrogen peroxide. #Weight marker (molecular weight in kDa): Amersham™ ECL™ Rainbow™ Marker - Full range, 12 to 225 kDa; catalogue number: RPN800E. The densitometry analysis was performed using the Image J program.

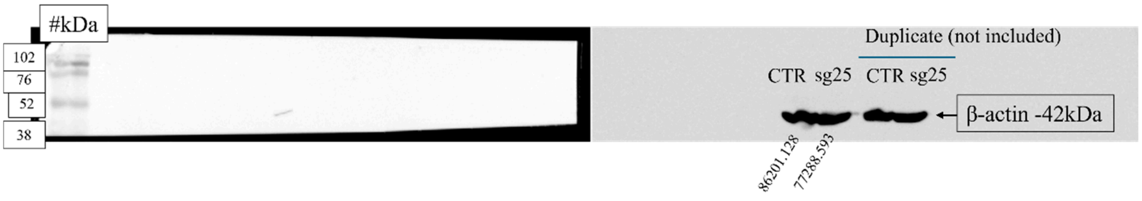

Figure S14. Western blot membrane of β-Actin (~42 kDa) protein detected with mouse anti-β-Actin (C4; 1:1000; Santa cruz, CA, USA) antibody diluted with 5% nonfat dry milk in Tris-buffered saline 0.1% Tween-20. Gel-separated proteins were transferred to nitrocellulose Hybond-ECL membranes (0.45 μm pore size; Amersham Biosciences, Little Chalfont, UK) by semi dry electroblotting at 30 V, overnight. Membrane was incubated with a horseradish peroxidase-conjugated secondary antibody (RPN1231-2ML; 1:4000; GE Healthcare, Little Chalfont, UK) and developed with luminol and p-coumaric acid (Sigma) reagents in the presence of hydrogen peroxide. #Weight marker (molecular weight in kDa): Amersham™ ECL™ Rainbow™ Marker - Full range, 12 to 225 kDa; catalogue number: RPN800E. The densitometry analysis was performed using the Image J program.

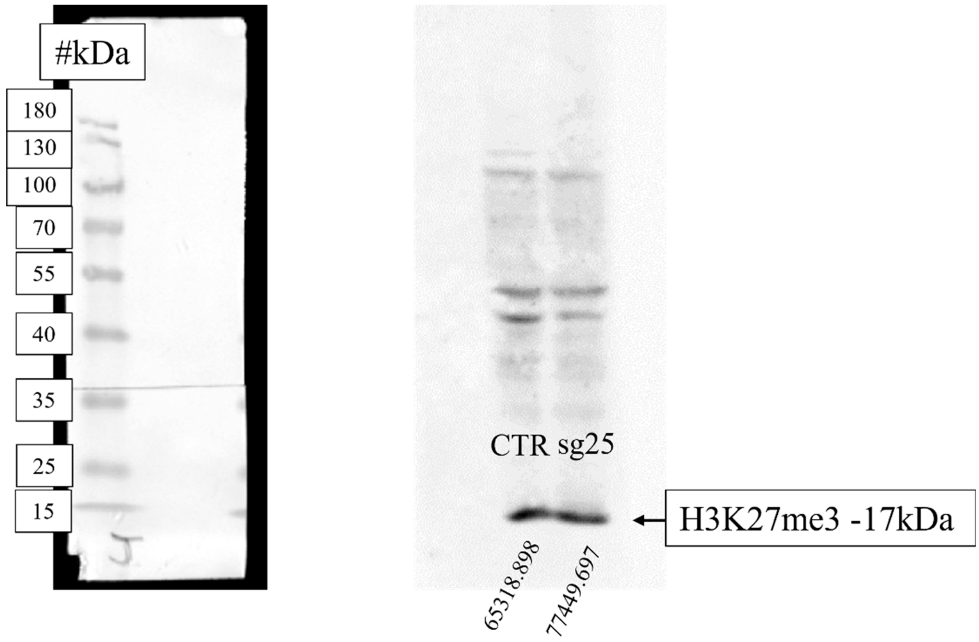

Figure S15. Western blot membrane of Tri-Methyl-Histone H3 (Lys27) (~17 kDa) protein detected with rabbit anti-Tri-Methyl-Histone H3 (Lys27) (C36B11; 1:1000; Cell signaling, Massachusetts, EUA) antibody diluted with 5% BSA in Tris-buffered saline 0.1% Tween-20. Gel-separated proteins were transferred to nitrocellulose Hybond-ECL membranes (0.2 µm pore size; Amersham Biosciences, Little Chalfont, UK) by semi dry electroblotting at 30 V, overnight. Membrane was incubated with a horseradish peroxidase-conjugated secondary antibody (RPN1231-2ML; 1:4000; GE Healthcare, Little Chalfont, UK) and developed with luminol and p-coumaric acid (Sigma) reagents in the presence of hydrogen peroxide. #Weight marker (molecular weight in kDa): Thermo Scientific™/PageRuler™ Prestained Protein Ladder, 10 to 180 kDa; catalogue number: 26616. The densitometry analysis was performed using the Image J program.

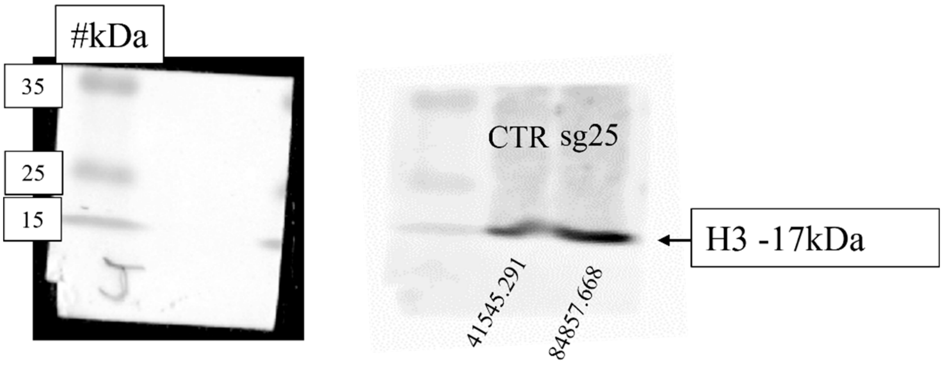

Figure S16. Western blot membrane of Histone H3 (~17 kDa) protein detected with rabbit anti-Histone H3 (D2B12; 1:2000; Cell signaling, Massachusetts, EUA) antibody diluted with 5% nonfat dry milk in Tris-buffered saline 0.1% Tween-20. Gel-separated proteins were transferred to nitrocellulose Hybond-ECL membranes (0.2 µm pore size; Amersham Biosciences, Little Chalfont, UK) by semi dry electroblotting at 30 V, overnight. Membrane was incubated with a horseradish peroxidase-conjugated secondary antibody (RPN1231-2ML; 1:4000; GE Healthcare, Little Chalfont, UK) and developed with luminol and p-coumaric acid (Sigma) reagents in the presence of hydrogen peroxide. #Weight marker (molecular weight in kDa): Thermo Scientific™/PageRuler™ Prestained Protein Ladder, 10 to 180 kDa; catalogue number: 26616. The densitometry analysis was performed using the Image J program.
