# Supplementary material for: Dual Effect of EZH2 Gene Editing with CRISPR/Cas9 in Lung Cancer
Source: Biology (Basel). 2026 Jan 29;15(3):251. doi: 10.3390/biology15030251 (PMC12896556; doi:10.3390/biology15030251)
Supplement: Supplementary file 1 [file biology-15-00251-s001.zip › Supplementary Figures S17-S20.pdf]

Supplementary Materials

A

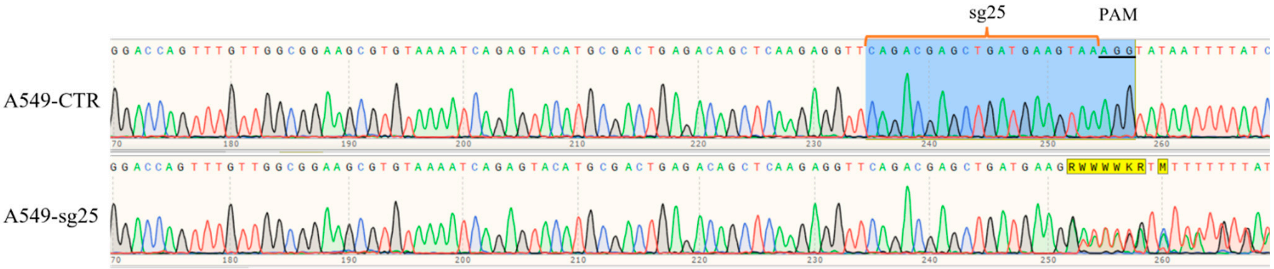

B

```
EZH2 WT     25 RLRQLKRFRRADEVKSMFSSNRQKIL 50
EZH2 -9     25 RLRQLKRFRRADEEYV*FQSSENFGK 50
EZH2 +1     25 RLRQLKRFRRADEGKEYV*FQSSENF 50
```

Figure S17. EZH2 gene editing with CRISPR/Cas9. (**A**) Raw sanger sequencing data alignment for *EZH2* gene used in SeqScreener Gene Editing. The blue highlighted region in A549-CTR indicates the region targeted by sgRNA25 and PAM sequence is underlined. (**B**) Alignment of amino acids changes after gene editing in A549-sg25 cells, showing the two most frequent editing events (9 nucleotide deletion and 1 nucleotide addition) and the insertion of stop codons (*) in edited cells. Sg: single guide; CTR: control; PAM: protospacer adjacent motif; *: premature stop codon.

A

WDR7

A549-CTR

A549-sg25

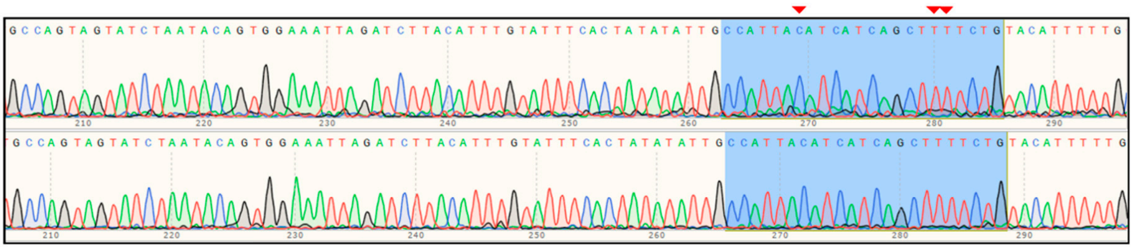

```
sg25           3' GGAAATGAAGTAGTCGAGCAGAC 5'
                  |||||||||||||||||||||||
Target EZH2    5' CCTTTACTTCATCAGCTCGTCTG 3'
WDR7 CTR       5' CCATTACATCATCAGCTTTTCTG 3'
WDR7 sg25      5' CCATTACATCATCAGCTTTTCTG 3'
```

B

DCLK

A549-CTR

A549-sg25

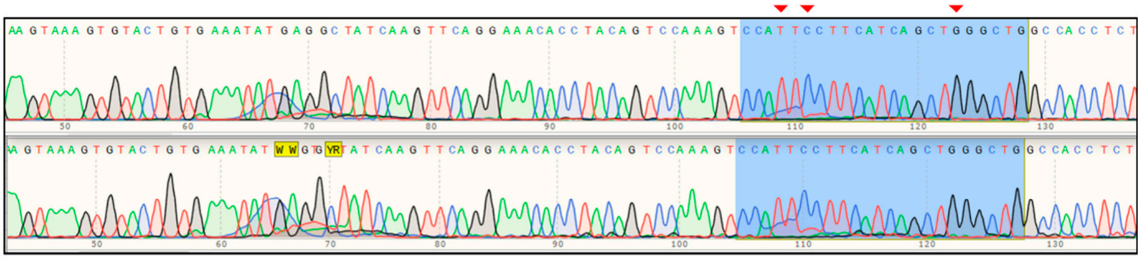

```
sg25           3` GGAAATGAAGTAGTCGAGCAGAC 5`
                  |||||||||||||||||||||||
Target EZH2    5' CCTTTACTTCATCAGCTCGTCTG 3'
DCLK CTR       5' CCATTCCTTCATCAGCTGGGCTG 3'
DCLK sg25      5' CCATTCCTTCATCAGCTGGGCTG 3'
```

Figure S18. Off-targets sequencing. Raw sanger sequencing data alignment for *WDR7* and *DCLK* genes, predicted off-target coding-genes in the ChopChop algorithm. (**A**) The panel shows the alignment of samples electropherogram and red arrowhead indicates potential off-target site of sgRNA25 in *WDR7* gene. Highlighted in blue, we indicate the potential target of sg25. In the below panel, we aligned sequences of A549-CTR and A549-edited cells showing no off-targeting in *WDR7* gene. (**B**) The panel shows the alignment of samples electropherogram and red arrowhead indicates potential off-target site of sgRNA25 in *DCLK* gene. Highlighted in blue, we indicate the potential target of sg25. In the below panel, we aligned sequences of A549-CTR and A549-edited cells showing no off-targeting in *DCLK* gene.

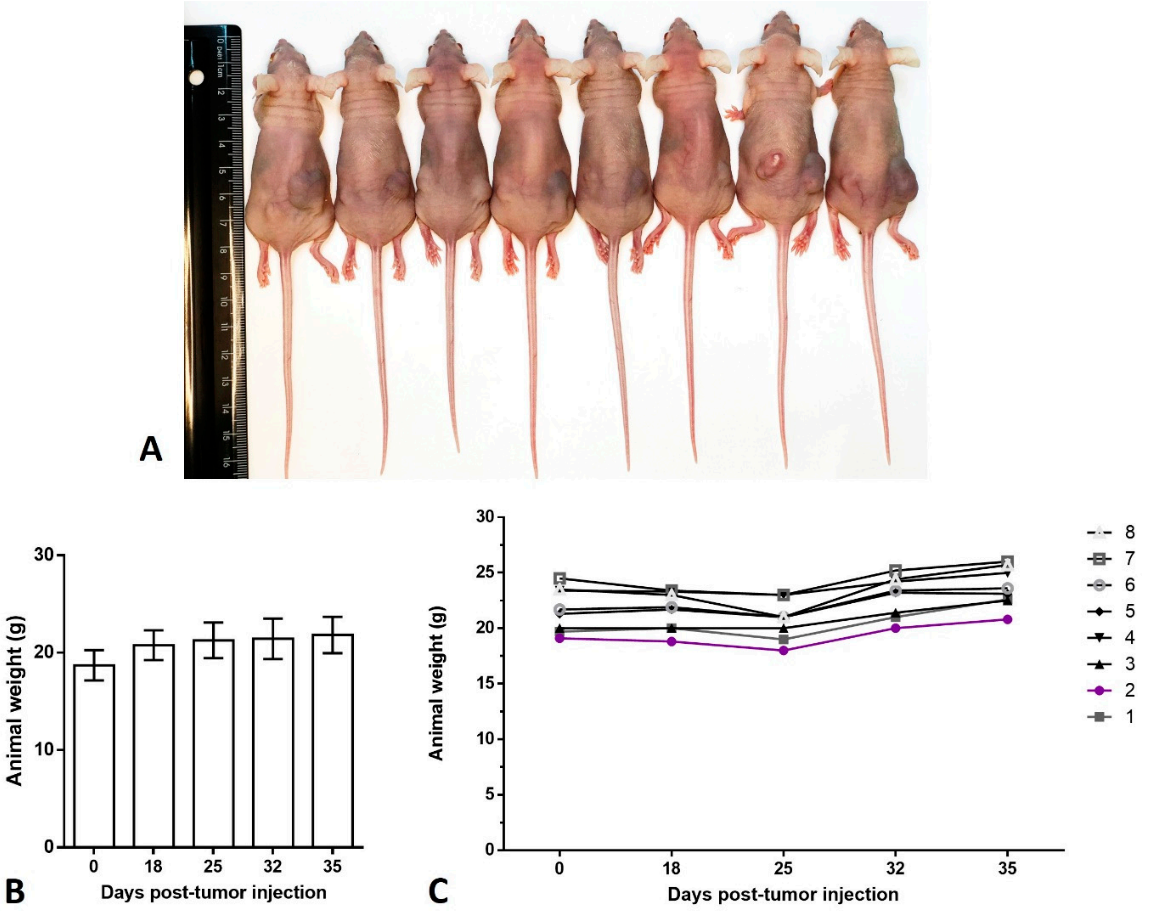

Figure S19. Mice weight monitoring and endpoint image after tumor xenotransplant (A): Endpoint image of the nude mice injected with A549-CTR cells (in the left flank) and A549-sg25 cells (in the right flank) (*n* = 8) at day 35. (B) Mean animal weight monitored along the 35-days period of experiment. (C) individual animal weight monitored along the 35-days period of experiment.

In vitro sequencing			indel	
36	**DEVKSMFSSNRQKILERTEILNQEWKQRRIQPVHILTSVSSLRGT**	80	**Wt**	**%sequences**
36	DEEYV*FQSSENFGKNGNLKPRMETAKDTACAHPDFCELIARD*G	80	**-9**	**40,98%**
36	DEXKEYV*FQSSENFGKNGNLKPRMETAKDTACAHPDFCELIARD	80	**+1**	**36,62%**
36	DEERVCLVPIVRKFWKERKS*TKNGNSEGYSLCTS*LL*AHCAGL	80	**-1**	**3,46%**
36	DEEYV*FQSSENFGKNGNLKPRMETAKDTACAHPDFCELIARD*G	80	**-5**	**3,15%**

Tumor sequencing			indel	
36	**DEVKSMFSSNRQKILERTEILNQEWKQRRIQPVHILTSVSSLRGT**	80	**Wt**	**%sequences**
36	DEERVCLVPIVRKFWKERKS*TKNGNSEGYSLCTS*LL*AHCAGL	80	**-1**	**48,34%**
36	DE--SMFSSNRQKILERTEILNQEWKQRRIQPVHILTSVSSLRGT	80	**-6**	**36,08%**
36	DEV-SMFSSNRQKILERTEILNQEWKQRRIQPVHILTSVSSLRGT	80	**-6**	**8,91%**
36	DEEYV*FQSSENFGKNGNLKPRMETAKDTACAHPDFCELIARD*G	80	**-9**	**2,43%**

Figure S20. Alignment of amino acid changes after *EZH2* gene editing with sgRNA25. In vitro sequencing was performed in the gDNA of A549-sg25 cells prior to injection into nude mice (Table S4), showing prevalence of nonsense alterations in DNA sequence (*premature stop codon insertion). Then, a fragment of the resulting tumor from A549-sg5 was removed (at the endpoint of the experiment 35 days) and gDNA was sequenced (Table S6), showing nonsense alterations in DNA sequence but also missense alterations (loss of one or two amino acids).
