# Supplementary material for: Dual Effect of EZH2 Gene Editing with CRISPR/Cas9 in Lung Cancer
Source: Biology (Basel). 2026 Jan 29;15(3):251. doi: 10.3390/biology15030251 (PMC12896556; doi:10.3390/biology15030251)
Supplement: Supplementary file 1 [file biology-15-00251-s001.zip › Supplementary Tables S1-S6.pdf]

Supplementary Materials

Table S1. Lung cancer cell lines used and culture conditions.

Cell line	Histology	Gender	Genetic driver	Culture medium	RRIDs
A549	LUAD	Male	$KRAS^{G12S}$, $STK11^{Q37*}$	DMEM + 10% FBS	CVCL_0023
Calu3	LUAD	Female	$KRAS^{G13D}$, $P53^{M237I}$	DMEM + 10% FBS	CVCL_0609
H2122	LUAD	Female	$KRAS^{G12C}$, $P53^{Q16L, C176F}$	RPMI1640 + 10% FBS	CVCL_1531

* FBS – fetal bovine serum, RRIDs – resource identification initiative

Table S2. List of primers used in this study.

	Sequence 5'-3'	Experiment
sgRNA 7 Fw	**CACCG**ACACGCTTCCGCCAACAAAC	cloning in PX459 plasmid
sgRNA 7 Rv	**AAAC**GTTTGTTGGCGGAAGCGTGT**C**	cloning in PX459 plasmid
sgRNA 18 Fw	**CACCG**TGCGACTGAGACAGCTCAAG	cloning in PX459 plasmid
sgRNA 18 Rv	**AAAC**CTTGAGCTGTCTCAGTCGCA**C**	cloning in PX459 plasmid
sgRNA 25 Fw	**CACCG**CAGACGAGCTGATGAAGTAA	cloning in PX459 plasmid
sgRNA 25 Rv	**AAAC**TTACTTCATCAGCTCGTCTG**C**	cloning in PX459 plasmid
EZH2 Fw	ATTTAACCATGCAGCACAAATG	DNA sequencing of target region
EZH2 Rv	CAGATCAAGAACCTAAGCTTCCA	DNA sequencing of target region
WDR7 FW	AAGCATGTGTTTCAGGGGCT	sg25 off-targeting DNA sequencing
WDR7 RV	TCTGTGTCTTCCCCTGTCCT	sg25 off-targeting DNA sequencing
DCLK3 FW	GAAGCAGGGTCGAGGTGAAA	sg25 off-targeting DNA sequencing
DCLK3 RV	GATGGCTGGAGAGTGGCTT	sg25 off-targeting DNA sequencing
RPL19 FW	TCTCATGGAACACATCCACAA	qPCR
RPL19 RV	TGGTCAGCCAGGAGCTTCTT	qPCR
ACTB FW	CATGTACGTTGCTATCCAGGC	qPCR
ACTB RV	CTCCTAATGTCACGCACGAT	qPCR
NKX2-1 FW	GCCGAAAAGAAAGAGCTGTGC	qPCR
NKX2-1 RV	ACCAGATCTTGACCTGCGTG	qPCR
CDH1 FW	TACCCTGGTGGTTCAAGCTG	qPCR
CDH1 RV	ACCTGACCCTTGTACGTGGT	qPCR
ZEB1 FW	GATGACCTGCCAACAGACCA	qPCR
ZEB1 RV	GCCCTTCCTTTCCTGTGTCA	qPCR
ZEB2 FW	AGTGTGCCCAACCATGAGTC	qPCR
ZEB2 RV	TCCTTCATTTCTTCTGGACCATC	qPCR
GATA5 FW	CCTCTTTCCGCATTGCAAGA	qPCR
GATA5 RV	TTCCGTGTCTGGATGCTTTC	qPCR
FOXA2 FW	CTGGTCGTTTGTTGTGGCTG	qPCR

FOXA2 RV	GGAGGAGTAGCCCTCGG	qPCR
SFTPA2 FW	GCCTGAAAAGAAGGAGCAG	qPCR
SFTPA2 RV	GGCTTCCAACACAAACGTCC	qPCR
SFTPB FW	AGCCATGATTCCCAAGGGTG	qPCR
SFTPB RV	ATTCTCCTGTCGGCGACCTT	qPCR
SFTPC FW	AACACACGGAGATGGTTCTGG	qPCR
SFTPC RV	GGCCTGGAAGTTGTGGACTTT	qPCR
SCGB1A1 FW	AAGCATCATTAAGCTCATGGAAAA	qPCR
SCGB1A1 RV	GTGGACTCAAAGCATGGCAG	qPCR
CBX2 FW	GCGGCTGGTCCTCCAAACA	qPCR
CBX2 RV	CTTGCCTCTCTTCCGGTTCT	qPCR
RING1 FW	TTGAGTCCCAGCATTGAGG	qPCR
RING1 RV	CCACTCATCGTTGTGGTCTG	qPCR
SUZ12 FW	GAAGCCGAAAATGGAGCACG	qPCR
SUZ12 RV	GTTCTGGAGTTTCGATGAGACAT	qPCR
EZH1 FW	GTTCTGGAGTTTCGATGAGACAT	qPCR
EZH1 RV	TGGAGGTAGGGGGATTTGGT	qPCR
EED FW	TGGATTTCTGGCAAAAGATGCT	qPCR
EED RW	TATCGAAGTCGATCCCAGCG	qPCR
pri-mir-200c FW	CTCGTGATCAGCGACCCAG	qPCR
pri-mir-200c RV	AGGGGCCTCCATCATTACCC	qPCR

* FW – forward, RV – reverse

Table S3. Validation of CRISPR/Cas9-mediated *EZH2* gene editing with sgRNA25 in A549 using the SeqScreener Gene App.

Contribution	DNA Sequence	Indel Size
40.98%	CGACTGAGACAGCTCAAGAGGTTCAGACGAGCTGATGAAG---------AATTTTATCTTTTGTGAAAATG	-9
36.62%	CGACTGAGACAGCTCAAGAGGTTCAGACGAGCTGATGAAG+TAAAGGTATAATTTTATCTTTTGTGAAAAT	1
3.54%	CGACTGAGACAGCTCAAGAGGTTCAGACGAGCTGATGAAGTAAAGGTATAATTTTATCTTTTGTGAAAATG	0
3.46%	CGACTGAGACAGCTCAAGAGGTTCAGACGAGCTGATGAAG-AAAGGTATAATTTTATCTTTTGTGAAAATG	-1
3.15%	CGACTGAGACAGCTCAAGAGGTTCAGACGAGCTGATGAA-----GGTATAATTTTATCTTTTGTGAAAATG	-5
2.94%	CGACTGAGACAGCTCAAGAGGTTCAGACGAGCTGATGAAG+++++TAAAGGTATAATTTTATCTTTTGTGA	5
1.70%	CGACTGAGACAGCTCAAGAGGTTCAGACGAGCTGATGAA------GTATAATTTTATCTTTTGTGAAAATG	-6
1.57%	CGACTGAGACAGCTCAAGAGGTTCAGACGAGCTGATGA---------ATAATTTTATCTTTTGTGAAAATG	-9
1.21%	CGACTGAGACAGCTCAAGAGGTTCAGACGAGCTGATG----AAAGGTATAATTTTATCTTTTGTGAAAATG	-4
1.14%	CGACTGAGACAGCTCAAGAGGTTCAGACGAGCTGATGAAG----------ATTTTATCTTTTGTGAAAATG	-10
0.99%	CGACTGAGACAGCTCAAGAGGTTCAGACGAGCTGATGAAG----GGTATAATTTTATCTTTTGTGAAAATG	-4
0.97%	CGACTGAGACAGCTCAAGAGGTTCAGACGAGCTGATGAAG---AGGTATAATTTTATCTTTTGTGAAAATG	-3
0.59%	CGACTGAGACAGCTCAAGAGGTTCAGACGAG---------TAAAGGTATAATTTTATCTTTTGTGAAAATG	-9
0.45%	CGACTGAGACAGCTCAAGAGGTTCAGACGAGCTGATGAAG++++++++++TAAAGGTATAATTTTATCTTT	10
0.27%	CGACTGAGACAGCTCAAGAGGTTCAGACGAGCTGATGAAG-------ATAATTTTATCTTTTGTGAAAATG	-7
0.21%	CGACTGAGACAGCTCAAGAGGTTCAGACGAGCTGAT----TAAAGGTATAATTTTATCTTTTGTGAAAATG	-4
0.15%	CGACTGAGACAGCTCAAGAGGTTCAGACGA----------TAAAGGTATAATTTTATCTTTTGTGAAAATG	-10
0.06%	CGACTGAGACAGCTCAAGAGGTTCAGACGAGCTGATGAAG+++TAAAGGTATAATTTTATCTTTTGTGAAA	3

Table S4. List of antibodies and dilutions for protein expression analysis.

Antibody	Source	Dilution	Application
mouse anti-E-Cadherin (4A2)	Cell signaling	1:1000	WB
rabbit anti-EZH2 (D2C9)	Cell signaling	1:1000	WB
mouse anti-Vimentin (RV202)	Santa cruz	1:1000	WB
mouse anti-β-Actin (C4)	Santa cruz	1:1000	WB
rabbit anti-EED (E4L6E)	Cell signaling	1:1000	WB
rabbit anti-EZH1 (D7D5D)	Cell signaling	1:1000	WB
rabbit anti-H3K27me3 (C36B11)	Cell signaling	1:1000	WB
rabbit anti-Histone H3 (D2B12)	Cell signaling	1:2000	WB
rabbit anti-β-Catenin (sc-7199)	Santa Cruz	1:1000	IHC

* WB – western blot, IHC – immunohistochemistry

Table S5. List of potential off-target genes of sgRNA25.

Genomic localization	N° of mismatches	Gene symbol	Observation	Sequence (including mismatches)
chr18:56722464	3	*WDR7*	Intronic	CCATTACaTCATCAGCTttTCTG
chr3:36731148	3	*DCLK3*	Intronic	CAGcCcAGCTGATGAAGgAATGG

Table S6. Validation of CRISPR/Cas9-mediated *EZH2* gene editing with sgRNA25 in A549 tumor *in vivo* using the SeqScreener Gene App.

Contribution	DNA Sequence	Indel Size
48.34%	CTGAGACAGCTCAAGAGGTTCAGACGAGCTGATGAAGTA-|AGGTATAATTTTATCTTTTGTGAAAATGAAT	-1
36.08%	CTGAGACAGCTCAAGAGGTTCAGACGAGCTGATG------|AGGTATAATTTTATCTTTTGTGAAAATGAAT	-6
8.91%	CTGAGACAGCTCAAGAGGTTCAGACGAGCTGATGAAGTA-|-----TAATTTTATCTTTTGTGAAAATGAAT	-6
2.43%	CTGAGACAGCTCAAGAGGTTCAGACGAGCTGATGAAG---|------AATTTTATCTTTTGTGAAAATGAAT	-9
1.80%	CTGAGACAGCTCAAGAGGTTCAGACGAGCT----------|AGGTATAATTTTATCTTTTGTGAAAATGAAT	-10
0.95%	CTGAGACAGCTCAAGAGGTTCAGACGAGCTGATGAA----|------AATTTTATCTTTTGTGAAAATGAAT	-10
0.83%	CTGAGACAGCTCAAGAGGTTCAGACGAGCTGATGAAGTAA|+AGGTATAATTTTATCTTTTGTGAAAATGAA	1
0.26%	CTGAGACAGCTCAAGAGGTTCAGACGAGCTGATG------|----ATAATTTTATCTTTTGTGAAAATGAAT	-10
0.19%	CTGAGACAGCTCAAGAGGTTCAGACGAGCTGATGAAGTAA|+++++++++++AGGTATAATTTTATCTTTTG	11
0.12%	CTGAGACAGCTCAAGAGGTTCAGACGAGCTGATGAAGTAA|+++++++++++++++++++AGGTATAATTTT	19
0.08%	CTGAGACAGCTCAAGAGGTTCAGACGAGCTGAT-------|--GTATAATTTTATCTTTTGTGAAAATGAAT	-9
